# The Isolated Thumb Domain of Acid‐Sensing Ion Channels Forms a Minimal Folding Unit Enabling Ligand Binding Studies

**DOI:** 10.1002/anie.202523977

**Published:** 2026-03-25

**Authors:** Biswa P. Mishra, Ben Cristofori‐Armstrong, Elena Budusan, Mimi Golder, Neville J. Butcher, Junyu Liu, Theo Crawford, Yanni K.‐Y. Chin, Anneka Pereira Schmidt, Xinying Jia, Taylor B. Smallwood, Richard J. Clark, Jan P. Wurm, Lachlan D. Rash, Mehdi Mobli

**Affiliations:** ^1^ Australian Institute for Bioengineering and Nanotechnology The University of Queensland St Lucia Queensland Australia; ^2^ School of Biomedical Sciences The University of Queensland St Lucia Queensland Australia; ^3^ Bruker Biospin Ettlingen Germany

**Keywords:** acid‐sensing ion channels, electrophysiology, NMR spectroscopy, peptide ligands, protein structure

## Abstract

Proton‐gated acid‐sensing ion channels (ASICs) are emerging therapeutic targets for ischemia‐related conditions such as stroke and myocardial infarction. Although structural data exist for ASIC1a, key aspects of ligand recognition and modulation remain unresolved. Using multidimensional solution‐state NMR spectroscopy, we show that the principal ligand‐binding region of ASICs, the thumb domain, forms an independently folded unit that at neutral pH adopts a native‐like conformation resembling the resting state of the channel. By integrating high‐resolution biophysical analyses of ligand binding to the isolated thumb domain with electrophysiological measurements on full‐length ASIC1a, we distinguished molecular interactions that determine binding affinity from those governing functional efficacy. This approach revealed that dynorphin A acts as a competitive antagonist of ASIC1a. Furthermore, NMR‐based p*K*_a_ determination of individual acidic residues demonstrated generally elevated values across the isolated thumb domain, supporting the presence of an extended acid‐sensing network rather than a single dominant pH sensor. These findings establish the isolated thumb domain as a powerful model for dissecting ASIC ligand interactions and pH sensitivity in solution, providing mechanistic insights and enabling structure‐based drug discovery of therapeutic modulators for ASICs and related ion channels.

## Graphical abstract

An independently folded thumb domain from acid‐sensing ion channels (ASICs) preserves ligand‐binding and proton‐sensing properties. NMR‐based interaction and p*K*_a_ analyses uncover how endogenous and exogenous peptides modulate ASICs, providing a simplified model for rational drug discovery targeting proton‐gated ion channels.

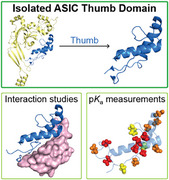

## 1 Introduction

The proton‐gated acid‐sensing ion channels (ASICs) are expressed in diverse tissues and cells where they play a pivotal role in detecting and responding to extracellular acidification. There are at least six functional isoforms (ASIC1a, 1b, 2a, 2b, 3, and 4) that exhibit unique biophysical and pharmacological properties and diverse roles in both physiological and pathological contexts [1]. Genetic studies have revealed their involvement in synaptic transmission, fear, and memory consolidation [2]. Beyond these physiological roles, the inhibition of ASICs by peptides has shown protective effects in rodent models of ischemic stroke, pain, and myocardial injury [3, 4, 5, 6]. Among the subtypes, ASIC1a and ASIC3 are the most pH‐sensitive, making them particularly attractive pharmaceutical targets. Consequently, there is considerable interest in developing potent and selective modulators for this ion channel family [1, 2].

**FIGURE 1:**
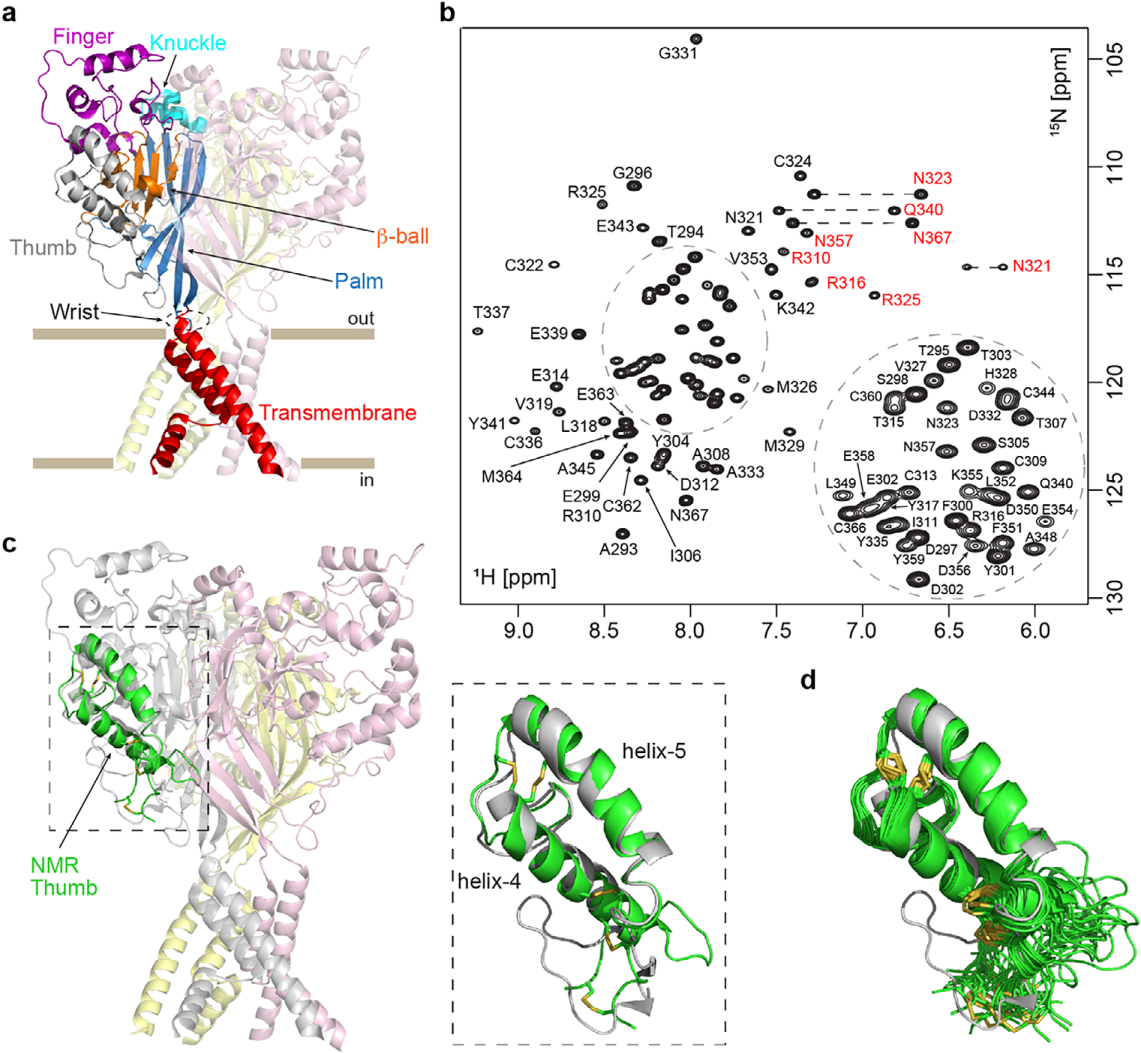
NMR structure of the isolated cASIC1a thumb domain. (a) Structure of the cASIC1a trimer with individual domains of one subunit color‐coded and labelled, while the other two subunits are shown in light pink and yellow (PDB: 5WKU) [9]. The lipid membrane region is represented by brown bars. (b) Fully assigned ^1^H–^15^N HSQC spectrum of the isolated cASIC1a thumb domain at 25 °C (pH 7). Assignments are labelled by residue name and sequence number from the full‐length cASIC1a sequence. Side chain correlations are highlighted in red, and asparagine/glutamine NH_2_ groups are connected by dashed lines. (c) Overlay of the NMR‐derived structure of the isolated thumb domain (green) with the resting‐state full‐length structure, with a zoomed‐in view showing the thumb domain (boxed inset). (d) Ensemble of 20 NMR structures of the isolated thumb (PDB: 7LIE), with disulfide bonds shown in yellow. The thumb domain is overlaid with the resting‐state thumb from the full‐length structure. The lack of NOESY‐derived distance restraints indicates the *N*‐ and *C*‐terminal regions and associated loop are dynamic in solution, resulting in poor overlay with the full‐length structure for these residues.

X‐ray crystallography and cryo‐EM studies revealed that functional ASICs are trimeric, with all available structures corresponding to the ASIC1a isoform [7, 8, 9]. Atomic resolution details of ligand binding to other ASIC subtypes remain largely unexplored, limiting our molecular‐level understanding of ligand selectivity and function. The extracellular region of a single ASIC subunit is modular, resembling a hand with distinct domains named the wrist, palm, knuckle, β‐ball, thumb, and finger (Figure 1a). The importance of the ASIC thumb is underscored by its critical role in proton‐sensing, with several amino acids in this domain playing a part in channel gating [9, 10, 11, 12]. Additionally, the thumb is a hotspot for modulation of channel function by endogenous and exogenous ligands [1]. Existing structures of ASIC1a in complex with peptide toxins show that they bind primarily to the thumb including a closed state with Mambalgin‐1, and several open states with PcTx1 or MitTx [13, 14, 15]. Such ligands have provided considerable insight into the structure and function of ASICs, further validating the thumb domain as an essential component in channel gating.

There is a scarcity of potent and isoform‐selective ASIC ligands, hindering both research and drug discovery efforts [1, 2]. Technical challenges in expressing correctly folded, full‐length channels of different subtypes and from different species further complicate ligand development. Moreover, screening the large extracellular region of ASICs can result in ligands binding to domains that are not directly involved in proton‐dependent gating. One strategy to overcome these limitations is to isolate channel domains known to be critical for ligand binding and channel gating, as has been done with the voltage sensor domain of voltage‐gated ion channels [16, 17, 18]. Given the ASIC thumb structure is stabilized by five disulfide bonds, and its architecture suggests a degree of structural independence from the channel core, this domain presents an attractive target for a channel deconstruction approach, enabling structural studies in isolation and with ligands under physiological solution conditions.

Here, we demonstrate that the ASIC1a thumb domain can be produced in isolation, retaining a native‐like fold, and ligand‐binding properties. This establishes the thumb domain as a minimal folding unit of ASICs, providing an accessible platform for detailed studies of ligand interactions and proton‐driven gating. By leveraging this approach, our work explores fundamental mechanisms of ASIC modulation, offering new avenues for research and future therapeutic development.

## 2 Results and Discussion

### 2.1 The Isolated ASIC Thumb Domain Retains Its Native Fold

The thumb domain of ASIC1a (residues 291–367 of the full‐length chicken channel; Uniprot: Q1XA76) lies post‐splice site and is therefore identical between ASIC1a and ASIC1b subtypes. Throughout this paper, we refer to the thumb as ASIC1a and use its residue numbering for consistency. The isolated chicken ASIC1a (cASIC1a) thumb domain was expressed in *E. coli* with a final yield of ∼0.75 mg/L of folded protein, sufficient for structural studies (Figure S1). To verify correct folding, uniformly labelled ^15^N/^13^C cASIC1a thumb was produced and used for subsequent structural characterization by NMR at pH 7. We used 2D (Figure 1b) and 3D heteronuclear NMR experiments for resonance assignment and extraction of NOE‐based structural restraints (Table S1). Structure calculations generated an ensemble of 20 3D solution structures of the isolated cASIC1a thumb (PDB: 7LIE), with well‐defined backbone and heavy‐atom RMSDs of 0.59 and 0.94 Å, respectively (for residues 305–354, see also Table S2).

Helix‐4, helix‐5, and the connecting loop in the isolated thumb are well ordered and in close agreement with that in the full‐length channel, aligning best with the resting state structure (RMSD of 1.42 Å to PDB ID 5WKU; Figure 1c) [9]. The *N*‐ and *C*‐termini are disordered, suggesting these regions are dynamic in solution (Figure 1d). The side chains of key acidic residues in both helices are oriented similarly to those in the full‐length channel, with the exception of D346, which is solvent‐exposed in the isolated domain but faces the channel body in the full‐length structure. Overall, our NMR structure confirms that the isolated thumb domain adopts a native‐like conformation independently of intra‐ASIC domain interactions, with the ligand binding interface largely intact.

To explore the applicability of our channel deconstruction approach to other ASIC subtypes and species, we expressed isolated thumb domains from rat ASIC1a (rASIC1a), rat ASIC3 (rASIC3), and human ASIC3 (hASIC3) (Figure S2). Stable globular folds were confirmed by well‐dispersed and narrow ^1^H‐NMR signals for all constructs, suggesting that the isolated thumb domain maintains structural integrity across ASIC subtypes. Conservation of the thumb domain structure across ASICs further highlights its potential as a research tool for probing ligand interactions.

### 2.2 Thumb Binding Shows DynA 2–17 is a Competitive Antagonist of BigDyn Function at ASIC1a

Although BigDyn is the most potent endogenous peptide modulator of ASIC1a reported [19, 20], no high‐resolution experimental structural data exists for this interaction. Interestingly, the circulating peptide fragments DynA and DynB, which derive from BigDyn, display either weak or no functional activity at ASICs. While some studies suggest that DynA exhibits similar activity to BigDyn at ASIC1a [20, 21], others report a >1000‐fold reduction in potency [19, 22], underscoring the need for further investigation. The thumb domain has been identified as a likely binding component for BigDyn [19, 22], but it remains unclear which parts of the channel interact with specific regions of BigDyn or its fragments, such as DynA.

**FIGURE 2:**
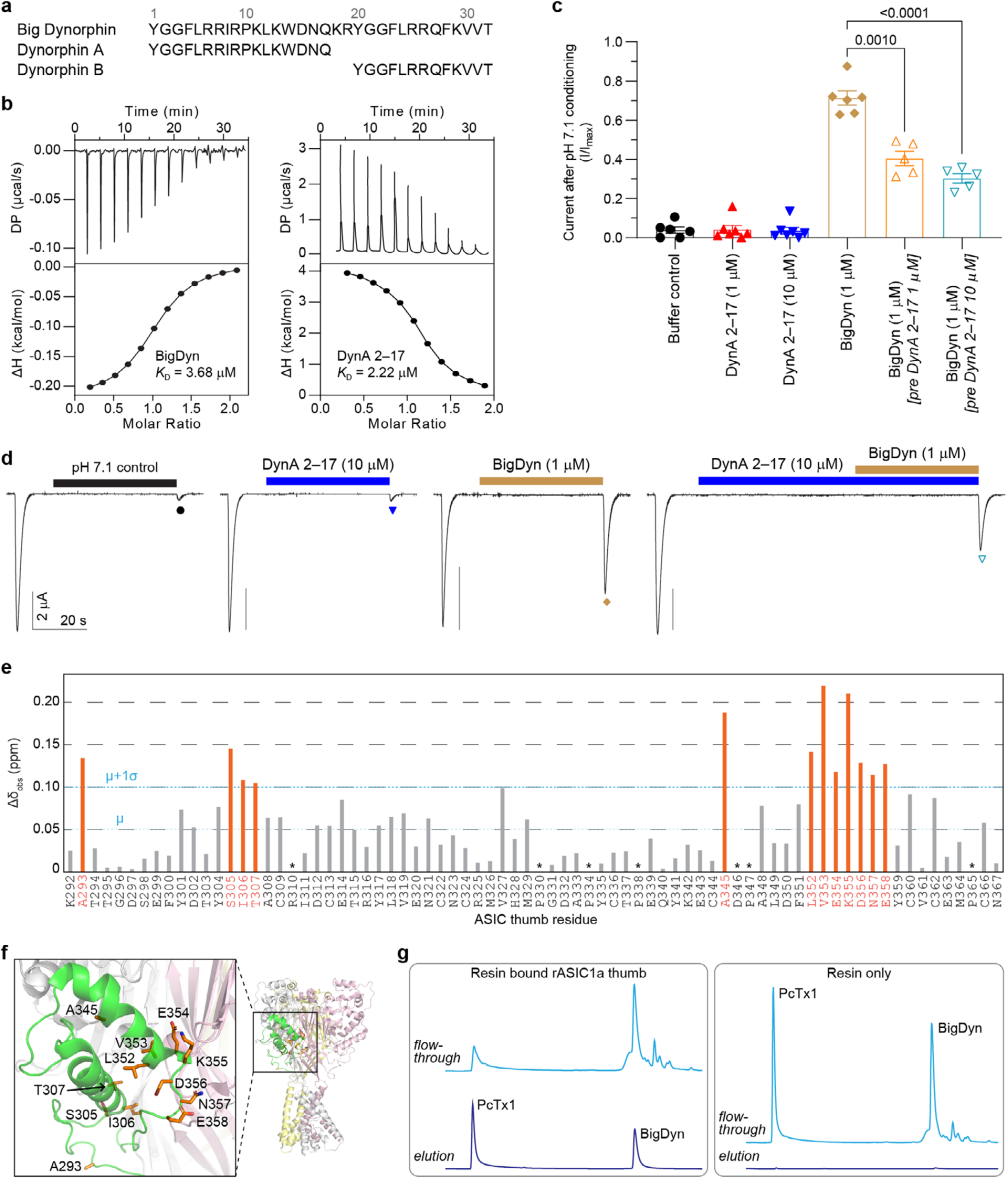
Structural and functional studies show that DynA 2–17 silently binds the ASIC1a thumb. (a) Amino acid sequences of Big Dynorphin (BigDyn), Dynorphin A (DynA), and Dynorphin B (DynB). DynA 2–17 is a single *N*‐terminal amino acid truncation of DynA. (b) ITC analysis of BigDyn (left) and DynA 2–17 (right) binding to the isolated cASIC1a thumb domain. Top panels show raw differential power (DP) data; bottom panels display normalized and integrated binding curves. (c) Summary of oocyte electrophysiology‐based functional competition studies showing that DynA 2–17 blocks BigDyn function at rASIC1a. Statistical analysis was performed using Welch's one‐way ANOVA with Dunnett's multiple comparisons test, with adjusted P‐values shown. Data are presented as mean ± SEM, *n* = 5–7 independent oocytes. (d) Example *Xenopus* oocyte current traces from functional competition studies. (e) Plot of NMR chemical shift changes (Δ*δ*) of the isolated cASIC1a thumb domain with the addition of DynA 2–17 at a 1:1 molar ratio. Significant shifts (mean + 1 SD) are shown in orange; asterisks (*) denote unobservable signals. (f) Resting‐state cASIC1a trimer structure (PDB: 5WKU) with the thumb domain of one subunit in green. Inset shows key residues with significant NMR shifts, labelled, and highlighted as orange sticks. (g) Peptide pulldown assay showing that the isolated rASIC1a thumb domain binds BigDyn and PcTx1 peptides. HPLC chromatograms display elution profiles from avidin‐agarose resin‐bound rASIC1a thumb (left) versus control resin (right). Peptides were applied from a 10 µM mixture in phosphate buffered saline, and bound peptides eluted using 0.1 M glycine pH 2.8.

To characterize the binding of dynorphins to the ASIC thumb domain, we first performed isothermal titration calorimetry (ITC) experiments with BigDyn and DynA 2–17 against the isolated cASIC1a thumb domain. Both peptides showed similar binding affinities, with dissociation constants (*K*_D_) of 3.68 µM for BigDyn and 2.22 µM for DynA 2–17 (Figure 2a, b and Table S3). Notably, the ITC profiles revealed clear differences in binding thermodynamics, with BigDyn displaying an exothermic profile, while DynA 2–17 showed an endothermic signature, suggesting distinct underlying enthalpic and entropic contributions to their interactions with the thumb. We next assessed whether these binding affinities correlated with functional outcomes using electrophysiology. Consistent with previous studies [19, 20, 22], BigDyn (1 µM) rescued rASIC1a from steady‐state desensitization (Figure 2c, d). In contrast, despite binding with a similar *K*_D_​ in our ITC experiments, 10 µM DynA 2–17 showed no functional activity under our electrophysiology experimental conditions, aligning with previous reports of reduced activity compared to BigDyn [19, 22]. These results suggest that while the *N*‐terminal segment of BigDyn is sufficient for binding, the *C*‐terminal segment likely contributes additional interactions that influence functional modulation. Furthermore, pre‐application of 1 µM DynA 2–17 inhibited the effect of 1 µM BigDyn on rASIC1a, and increasing the DynA 2–17 concentration to 10 µM enhanced this inhibitory effect (Figure 2c and d). These results reveal that although DynA 2–17 has no intrinsic functional activity at ASIC1a, it can bind to the thumb domain and act as an antagonist of BigDyn.

Next, we turned to NMR chemical shift mapping to gain structural insights into the dynorphin‐ASIC1a interaction, using isolated ^15^N‐cASIC1a thumb domain and unlabeled BigDyn or DynA 2–17 (Figure S3). Initial NMR experiments with BigDyn showed line broadening (Figure S3), suggesting binding kinetics in the µs‐ms time scale, due to chemical and/or conformational exchange. In contrast, DynA 2–17 binding occurred in the fast exchange regime, consistent with the micromolar *K*_D_ value observed by ITC. Mapping the chemical shift changes revealed significant perturbations in the thumb's helix‐5 region, particularly at residues A345, L352, V353, E354, K355, D356, N357, and E358 (Figure 2e). Additionally, several residues in the disordered region of the thumb also undergo considerable chemical shift perturbations (A293, S305, I306, and T307). Helix‐5 likely represents a true binding interface, as perturbations here were consistently observed during DynA 2–17 binding. In contrast, the functional relevance of the shifts in the disordered loop is harder to interpret, as our structure shows this region to differ from that in the intact channel, but may reasonably result from either change in the dynamics or structure of this region upon DynA 2–17 binding. These findings provide the first direct structural insights into the dynorphin‐ASIC1a binding interface through NMR, complementing existing crosslinking data and offering a detailed view of how DynA engages the thumb domain (Figure 2f). Together, these results suggest that the *N*‐terminal DynA segment of BigDyn binds to the lower thumb domain, including helix‐5, as mapped by our NMR titrations. This region overlaps with contacts identified in photo crosslinking studies using full‐length BigDyn [19, 22] (Figure S4), which additionally highlight interactions with the complementary subunit forming the acidic pocket and extending to the top of helix‐5. These additional contacts are likely made by the *C*‐terminal DynB segment of BigDyn, providing a structural rationale for the competitive antagonism by DynA 2–17.

To further explore the utility of the isolated ASIC thumb domain, we investigated its use in ligand pulldown assays. By immobilizing the isolated rASIC1a thumb domain onto resin, we successfully pulled down both BigDyn and PcTx1 (Figure 2g, S5). This was evidenced by distinct elution peaks for both ligands, whereas control resin without the thumb domain showed no such interactions. These results suggest that, the ASIC thumb domain could be a general and versatile tool for ligand screening in pulldown experiments. Beyond ASICs, the thumb domain also shows remarkable conservation across the wider DEG/ENaC family, as indicated by AlphaFold models and structural alignments, despite low sequence identity (Figures S6 and S7) [23]. This conservation demonstrates its potential as a tool for probing protein‐ligand interactions across DEG/ENaC channels, extending the utility of this approach beyond ASIC subtypes. Notably, our successful expression of the rat ASIC1a and rat and human ASIC3 thumbs suggests this system holds promise for future pulldown studies aimed at isolating potential endogenous ligands, including ASIC2 and ASIC4, for which little is known about endogenous modulators. Moreover, the ease of expressing thumb domains from different ASIC subtypes positions this system as a valuable tool for studying subtype‐specific ligand interactions, counter‐screening strategies, and drug discovery efforts aimed at modulating ASIC function in conditions that mimic either physiological or pathological contexts.

### 2.3 PcTx1 Interaction With the ASIC1a Thumb Recapitulates Full‐Length Channel Binding

**FIGURE 3:**
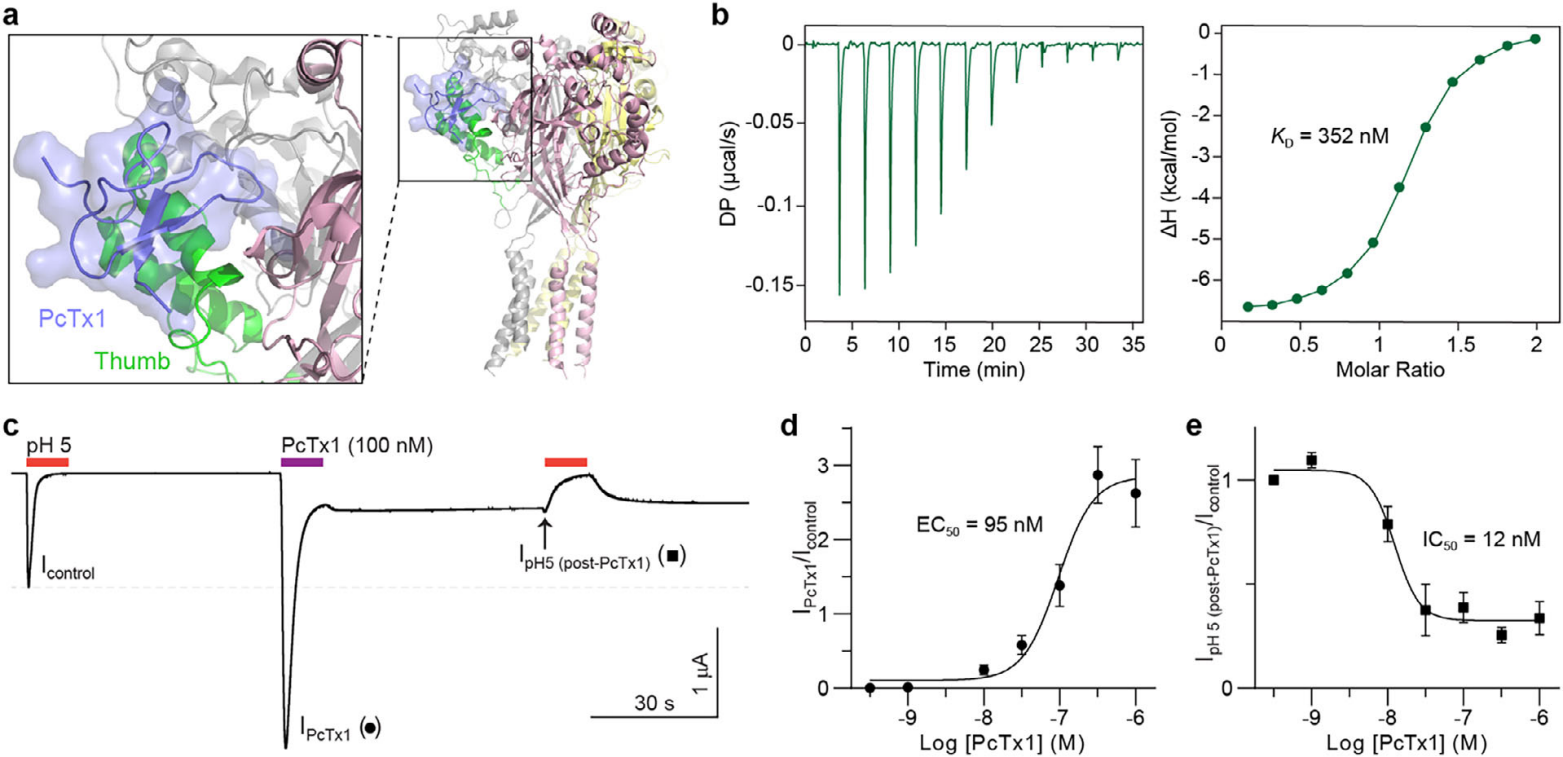
Interaction between PcTx1 and cASIC1a. (a) Co‐crystal structure of PcTx1 with cASIC1a showing the peptide binds predominantly to the thumb domain (PDB 4FZ0). (b) Binding of PcTx1 to the cASIC1a thumb measured by ITC at pH 7. (c) Example electrophysiology trace illustrating the transient activation of cASIC1a by PcTx1 (100 nM) at pH 7.5, followed by inhibition of pH 5‐induced current. (d) Concentration‐response curve for PcTx1‐mediated activation of cASIC1a at pH 7.5. (e) Concentration‐response curve for pH 5‐evoked current inhibition after application of different concentrations of PcTx1. Electrophysiology data are mean ± SEM and *n* = 5–7 independent oocytes.

PcTx1 is the prototypical ASIC1a modulating peptide. Functional and structural studies revealed that the thumb domain constitutes ∼70% of binding interactions [14, 24] (Figure 3a). Using ITC, we confirmed high affinity binding of PcTx1 to the isolated cASIC1a thumb at pH 7 with a *K*_D_ of 352 nM and a stoichiometry of 1 (Figure 3b). Binding is characterized by a favorable exothermic enthalpy at pH 7. To account for potential contributions from buffer ionization, control ITC experiments were performed in buffers with distinct ionization enthalpies. While the binding affinity was unchanged, the observed enthalpy varied between buffers, indicating coupling of complex formation to proton transfer. Analysis using (Δ*H_obs_* = Δ*H_bind_* + *n*Δ*H_ion_* yields an intrinsic, buffer‐independent binding enthalpy of ∼ ‐5.6 kcal mol^−1^ and a proton linkage term of n_H+_ ∼ ‐1.4, consistent with the net proton release upon complex formation (Figure S8, Table S4). These data indicate that proton coupling contributes to the measured enthalpy while preserving the underlying binding affinity. To contextualize thumb binding affinity, we measured PcTx1 activity at full‐length cASIC1a using two‐electrode voltage clamp electrophysiology in *Xenopus* oocytes. Notably, the pharmacology of PcTx1 at cASIC1a is not well characterized [14, 25]. Our electrophysiology experiments show that PcTx1, at pH 7.5, transiently activates cASIC1a with an EC_50_ of 95 nM (Figure 3c, d), followed by potent inhibition of pH 5‐induced currents (IC_50_ = 12 nM, Figure 3e). While the isolated thumb retains the primary binding determinants for PcTx1, the reduced binding affinity relative to full‐length channels highlights the importance of additional complementary face interactions for achieving full binding strength.

NMR chemical shift mapping revealed widespread perturbations in the isolated thumb domain upon PcTx1 binding, with the largest chemical shift changes observed for residues within the known binding interface (K342, F351 and K355) as well as residues in the helix‐4‐helix‐5 region (V327, A345 and V353), consistent with both direct ligand interactions and conformational rearrangements within the isolated thumb domain (Figure S9, S10a–c). In contrast, the titration performed using ^15^N‐labelled PcTx1 showed shifts primarily to the pharmacophore residues, aligning well with the co‐crystal structure [14], supporting a restricted and well‐defined pharmacophore loop in PcTx1 at the binding interface (Figure S10d, e). The more broadly dispersed perturbations observed in the thumb domain during the titration likely reflect inherent flexibility and dynamics of the thumb in solution. Residues distal to the primary PcTx1 contact site may exhibit chemical shift perturbations due to secondary effects propagated across the thumb upon PcTx1 binding. Overall, these findings demonstrate that NMR chemical shift mapping of the isolated thumb domain provides a global view of PcTx1 interactions, while highlighting its utility for identifying key binding surfaces and capturing ligand‐induced structural dynamics in solution.

**FIGURE 4:**
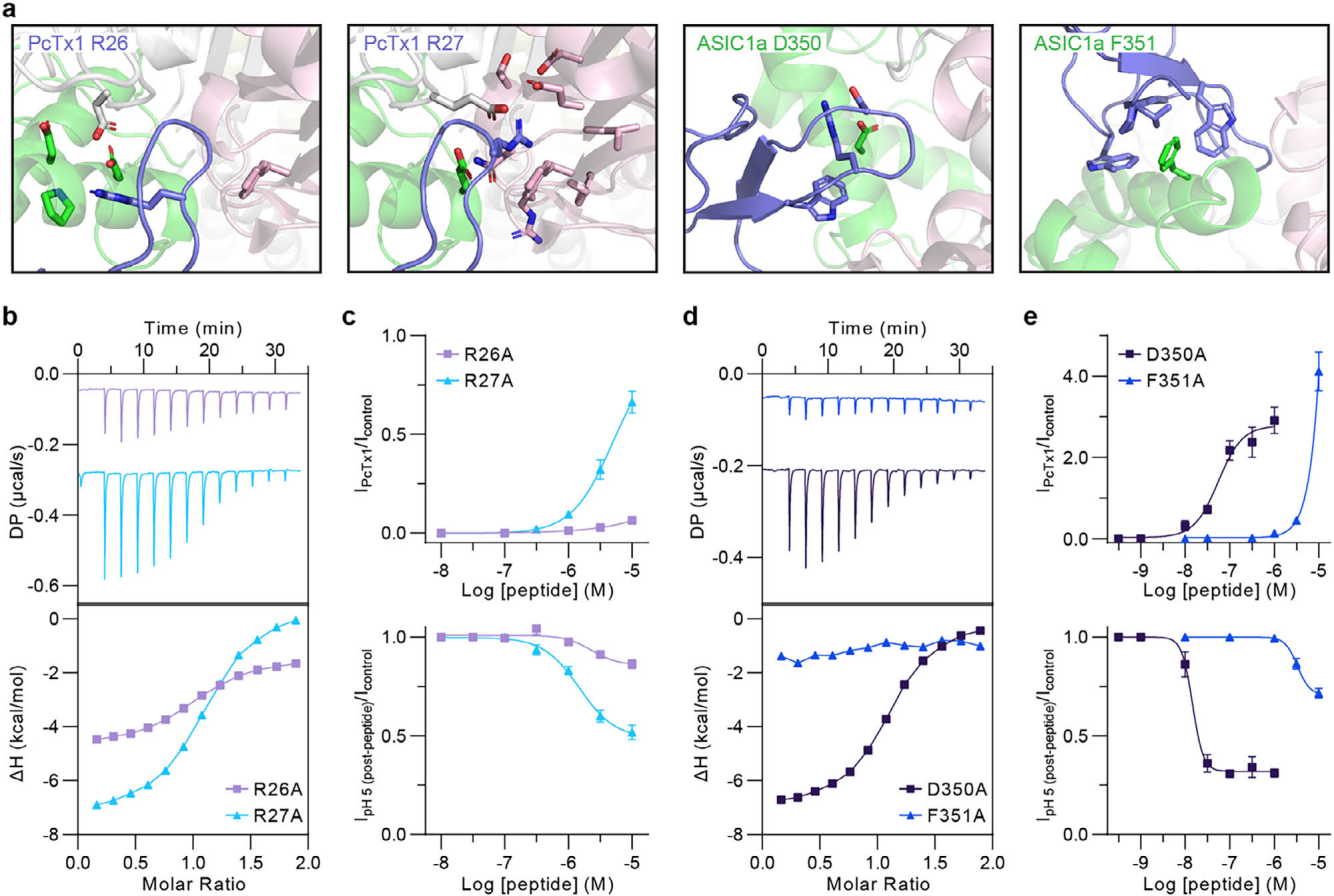
Binding interface between PcTx1 and cASIC1a. (a) Cartoon representation of PcTx1 (purple) bound to cASIC1a, showing interactions with two subunits at their interface. The thumb domain (green) belongs to the primary subunit (grey), while the complementary face subunit is shown in pink. Side chains of residues with an intermolecular distance of ≤5 Å of the mutated amino acid (labelled in the top left of each box) are shown to illustrate the interactions made by tested mutants. (b) ITC of PcTx1 R26A and R27A mutants with cASIC1a thumb. (c) Electrophysiology of PcTx1 R26A and R27A at wild type cASIC1a. (d) ITC of wild‐type PcTx1 with isolated cASIC1a thumb D350A and F351A equivalent mutations. (e) Electrophysiology of PcTx1 at cASIC1a D350A and F351A mutants. Electrophysiology data are mean ± SEM, and *n* = 5–7 independent oocytes. Thermodynamic properties and summary of replicates for ITC experiments is presented in Table S4, and electrophysiology concentration‐response summary is presented in Table S5. 1D NMR experiments for PcTx1 and cASIC1a thumb mutant variants showed that they maintained structural integrity (Figure S11).

While NMR chemical shift mapping identifies the general binding interface by detecting changes in the local chemical environment, it does not reveal which residues are energetically important for binding. To address this, we performed mutagenesis on both PcTx1 and the isolated thumb, followed by ITC and electrophysiology assays to dissect residue‐specific contributions to binding affinity and functional activity (Figure 4a). PcTx1 mutants R26A and R27A each exhibit approximately two‐fold reductions in binding affinity to the isolated thumb domain (*K*_D_ = 707 and 553 nM, respectively; Figure 4b, and Table S4), despite both residues forming extensive contacts with ASIC1a in the complex structure. In contrast, electrophysiological recordings at cASIC1a reveal severe losses in functional potency for both mutants (>50‐fold relative to wild type for both agonist and inhibitory effects; Figure 4c, Table S5). For R26A, which primarily disrupts interactions with thumb‐domain residues, this divergence between a small reduction in binding affinity and a pronounced loss of functional activity indicates that these thumb‐mediated contacts are particularly important for productive coupling of PcTx1 binding to channel modulation. R27A also shows a relatively small reduction in thumb‐binding affinity and displays a similarly severe loss of functional activity. This is consistent with R27's major contacts involving complementary face residues in the full‐length channel, which are absent in the isolated thumb system. These results indicate that the reduced functional activity is not primarily due to a loss of binding, but rather reflects impaired coupling of binding to channel modulation, likely mediated by complementary face interactions. These two PcTx1 mutants highlight the utility of the isolated thumb system, in combination with full‐length channel electrophysiology, in differentiating thumb‐specific contributions to ligand binding and the coupling of this binding to channel function.

On the cASIC1a thumb, we targeted residues equivalent to D350 and F351 to assess their roles in PcTx1 binding and functional activity. Although NMR titrations showed no significant perturbations for D350, the PcTx1‐ASIC1a complex structure suggests it may make multiple contacts. In agreement with the NMR data, the D350A mutant thumb retained binding affinity (Figure 4d; *K*_D_ 336 nM, Table S4) and functional efficacy comparable to wild‐type in full‐length channels (Figure 4g; agonist EC_50_ 55 nM and inhibition IC_50_ 15 nM). In contrast, F351 shows one of the larger chemical shift perturbations upon PcTx1 binding, and consistent with this, no measurable binding was observed in ITC measurements between PcTx1 and cASIC1a F351A thumb (Figure 4d), and this mutation severely diminished functional activity (Figure 4e). Unlike the PcTx1 mutants, where functional activity was disrupted despite preserved binding affinity, the F351A mutation caused a substantial loss in both binding affinity and functional efficacy. This indicates that the diminished functional activity observed for F351A is directly attributable to its impaired binding affinity.

### 2.4 pH‐Dependent Effects on PcTx1 Binding

**FIGURE 5:**
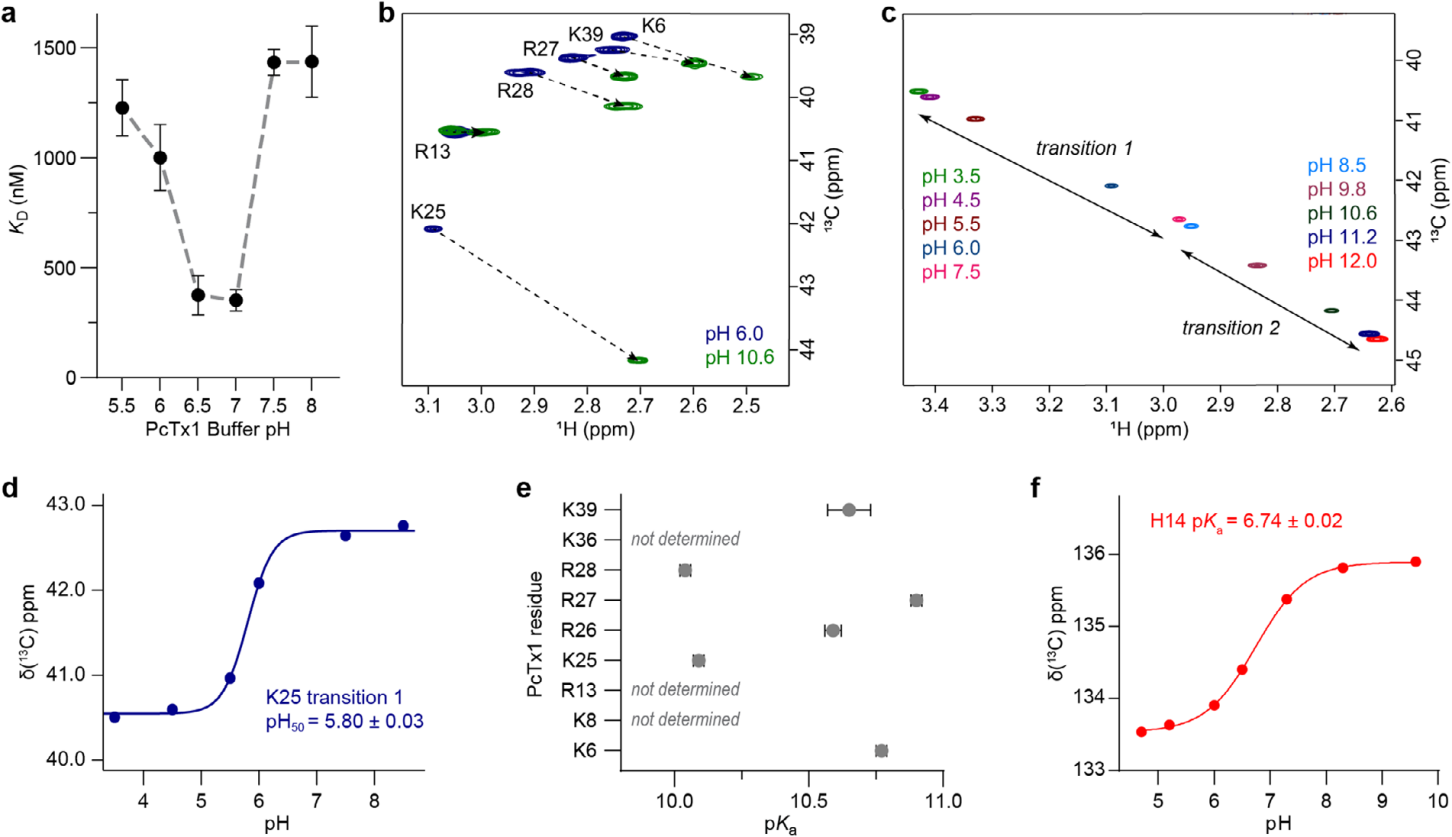
pH‐dependence of PcTx1 binding to ASIC1a. (a) ITC measurements of PcTx1 binding to the cASIC1a thumb at various pH values, showing a bell‐shaped binding profile centred around pH 7. The grey dashed line connects data from separate experiments at different pH values and does not represent a fitted model. Data are mean ± SEM, and *n* = 3–6 independent ITC experiments. (b) Superimposed band selective ^1^H‐^13^C HSQC spectra of PcTx1 at 25 °C; pH 6.0 (blue) and pH 10.6 (green). The signals appear as singlets in the ^13^C dimension as the pulse sequence includes a selective pulse to refocus the *J*‐coupling to the attached ^13^C atom and the coupling to the attached nitrogen is removed using ^15^N decoupling during acquisition. (c) Same as b, but showing all titration points for K25, revealing two significant transitions: one in the pH range of 3.5–7.5, and another from 8.5–12.0. The second shift reflects side chain protonation, with a calculated p*K*_a_ of 10.09 ± 0.02. (d) Fitting the Henderson–Hasselbalch equation to the first chemical shift transition of K25, occurring in the range of pH 3.5–7.5. (e) Experimentally determined p*K*_a_ values for lysine and arginine residues of PcTx1 based on NMR chemical shifts. Error bars represent the standard error of the fitted p*K*_a_ values obtained from nonlinear regression to the Henderson–Hasselbalch equation. (f) ^13^C chemical shift of ^13^C_ε_ of H14 in PcTx1 plotted against pH to calculate the p*K*_a_.

PcTx1 binding to ASIC1a is known to be pH‐sensitive, following a bell‐shaped curve with weaker binding at pH values above and below pH 7 [26, 27]. To determine whether this pH sensitivity is retained in the isolated thumb domain, we conducted ITC at various pH values (5.5, 6.0, 6.5, 7.0, 7.5, and 8.0). The strongest binding was observed at pH 6.5 and 7.0, with a notable decrease in affinity at more acidic and basic pH values (∼1 kcal/mol; Figures 5a, S12, and Table S6), consistent with published findings.

To investigate pH‐dependent effects on PcTx1, we measured the p*K*_a_ of lysine and arginine residues using a selective ^13^C HSQC experiment with selective decoupling of the adjacent ^13^C and ^15^N resonances (Figures 5b–e, S13). These experiments monitored chemical shifts of the terminal carbon atoms of lysine (^13^C_ε_) and arginine (^13^C_δ_) side chains, which are sensitive to the protonation state of the neighboring ionizable group. Figure 5b illustrates the changes in the ^13^C HSQC spectra of PcTx1 as the pH is increased from 6.0 (blue) to 10.6 (green). The ^13^C resonances of K8 and K36 were too weak to detect and could not be assigned, while the chemical shift changes in R13 were too small to allow reliable fitting; consequently, these residues have been excluded from analysis. For the remaining arginine and lysine residues, fitting of the data to the Henderson–Hasselbalch equation shows a clear transition in the expected range (p*K*_a_ >10; see Figures 5c, d, S13). Interestingly, K25 displayed two distinct transitions: one near pH ~5.8, suggesting a structural rearrangement, and a second at pH ∼10.1, consistent with its intrinsic p*K*_a_ (Figure 5c–e). The p*K*_a_ of H14 in PcTx1 was determined by monitoring the imidazole ^13^C_ε_─^1^H_ε_ chemical shifts as a function of pH, and fitting of the data showed a transition in the expected range (p*K*_a_ 6.7, see also Figure 5f), consistent with the reduced binding observed by ITC above pH 7. These observations highlight an important consideration for ASIC research: while pH changes are routinely used to manipulate channel state and activity, such changes can also impact ligand behavior, influencing both binding affinity and functional outcomes. Understanding these pH‐dependent effects is critical for accurately interpreting ASIC‐ligand interactions and designing experiments that account for these nuances.

While these results provide insights into the molecular basis for the loss of PcTx1 affinity at high pH, the reduced binding at low pH is likely driven by protonation of acidic residues in the thumb domain. Protonation of acidic residues in the thumb is particularly relevant, as these residues contribute to channel proton sensitivity but have, until now, only been estimated using in silico approaches [28, 29]. Therefore, we next set out to experimentally determine the pH sensitivity of the acidic residues in the thumb.

### 2.5 Site‐Specific p*K*_a_ Determination of Acidic Thumb Residues

To further understand the pH‐dependent structural changes in the ASIC1a thumb, we conducted NMR chemical shift mapping experiments on the isolated cASIC1a thumb domain. Chemical shift mapping of backbone amides provides a measure of structural changes in the protein as a consequence of acidification, allowing us to pinpoint specific residues sensitive to protonation or deprotonation events (Figures S14, S15a). The ^15^N HSQC spectra measured at pH 7 and 4 overlay poorly, consistent with a conformational change observed previously in the thumb domain upon acidification (Figure S14). These titrations further showed that, backbone amides in different regions of the thumb experience signal broadening at different pH ranges (Figure S15b). In NMR, chemical or conformational exchange processes in the µs to ms timescale can cause such signal decay, referred to as intermediate exchange. Several residues in the helix‐4–helix‐5 loop appear well resolved at neutral pH, are then broadened beyond detection at ∼pH 5.5, before reappearing at even lower pH. By contrast, many residues in helix‐4 and helix‐5 appear sharpest at pH 5.5, and while slightly broadened at neutral pH they are not detectable at low pH (∼4). The residues in the N‐ and C‐termini of the thumb show a similar behavior but are still detectable at pH 4. The observed signal broadening in the helix‐4–helix‐5 loop is of interest as it occurs near the known pH required for channel activation. To probe if the signal broadening observed in this loop at pH 5.5 can be due to µs‐ms dynamics, we performed a ^15^N CPMG experiment at pH 6, where the resonances of interest are still detectable (Figure S15c and Table S7). The results support the presence of µs–ms timescale motion as a source of the observed signal loss. While a structural change in the bottom of helix‐5 can be observed in the open (low pH, e.g., PDB: 9E4G) and closed states (high pH, e.g., PDB: 9E4B), coupling of these changes to residues known to be important for pH sensitivity in the helix‐4–helix‐5 loop such as H328 is unclear, and could be related to the dynamics observed here.

**FIGURE 6:**
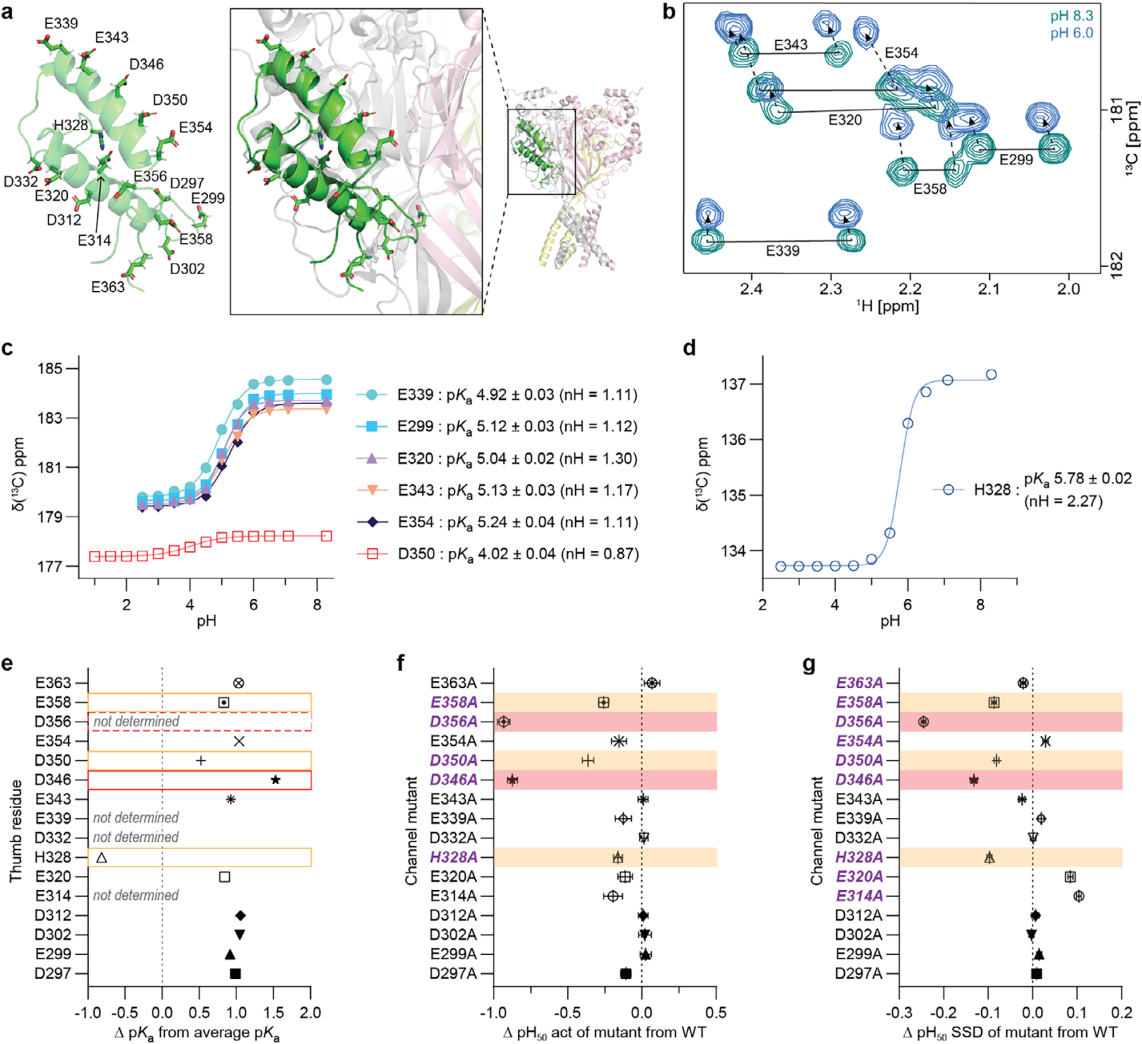
pH‐sensitivity of protonatable side chains on the cASIC1a thumb domain. (a) Resting state cryo‐EM cASIC1a trimer structure with each subunit colored in different light shades (PDB: 5WKU), overlaid with the NMR‐derived structure of the isolated cASIC1a thumb domain in green (PDB: 7LIE). Box shows the zoomed‐in view of the overlay, with acidic side chains on the isolated thumb shown as sticks. Left panel shows only the NMR thumb domain to highlight the stacking of acidic residues. (b) Superimposed 2D projections (^1^H and carboxyl ^13^C dimensions) of 3D HCaCO experiments, showing glutamate C_δ_ resonances from cASIC1a thumb at two different pH values: pH 8.3 in teal, pH 6.0 in blue. (c, d) ^1^H‐^13^C HSQC experiment tracking ^13^C chemical shifts and experimental titration curves for (c) selected glutamate and aspartate residues, and (d) H328. (e) Difference in p*K*_a_ values of thumb domain residues measured by NMR, shown relative to the average p*K*_a_ values typically observed for negatively charged amino acids [30]. The dashed line at 0.0 indicates no change in p*K*_a_ from the average value. D346 highlighted via a red box exhibits the largest deviations from typical p*K*_a_ values, in agreement with electrophysiology (see red shading in panels (f) and (g)). D356 (red dashed box) was undetectable in NMR titrations, suggesting potential conformational dynamics that might contribute to its role in proton sensitivity. Moderate p*K*_a_ shifts from average values observed for E358 and D350 (orange boxes) correspond to moderately impactful functional pH_50_ differences to wild type (see orange shading in panels (f) and (g)). (f, g) Difference in the measured pH_50_ of (f) activation and (g) steady‐state desensitization (SSD) for alanine mutated channels compared to wild type cASIC1a (data are presented as mean ± propagated SEM, calculated from independent WT and mutant pH_50_ values; *n* = 6–9 independent oocytes). Red shading highlights D356 and D346 as the most disruptive mutations to pH sensitivity, showing the largest deviations in both activation and SSD compared to wild‐type. Orange shading indicates moderately impactful residues like E358 and D350, which show deviations in both activation and SSD, though to a lesser extent. pH_50_ values were determined using oocyte electrophysiology (see Figure S17 and Table S8). Channel mutants where the label is bold, italicized, purple text indicates a significant difference (*p* < 0.05) in pH_50_ value from WT (Welch's one‐way ANOVA with Dunnett's multiple comparisons test).

To determine the order of protonation or the proton sensitivity of individual acidic residues on the isolated cASIC1a thumb, we monitored the side chain chemical shifts near the protonation sites using NMR spectroscopy. Specifically, we measured the acid dissociation constants (p*K*_a_’s) of aspartate (Asp, ^13^C_γ_), glutamate (Glu, ^13^C_δ_), and histidine (His, ^13^C_ε_) residues (Figures 6a, b, and S16a, b). Complete titration curves could be produced for the histidine and a majority of glutamic acid residues, however the aspartic acid residues were exchange broadened beyond detection below ∼pH 5 (Figures 6c, d, and S16c, d). This suggests that the protonation of the aspartic acid residues may be related to the exchange broadening seen in the backbone amides in the helix‐4‐helix‐5 loop. The p*K*_a_ values of Asp, Glu, and His were determined by fitting the chemical shift data to a modified Henderson–Hasselbalch equation to obtain the best‐fit p*K*_a_ value for each residue (Figure 6c–e). The modified equation includes a term for the Hill slope, but does not explicitly include terms for chemical shift changes due to protonation of nearby residues or conformational changes, which tend to be smaller than those arising from protonation of the measured carboxylic acid carbon (∼4 ppm). Where full titration curves could not be obtained (D297, D302, D312, D346, and E358), p*K*_a_ values were estimated by assuming a ^13^C chemical shift value change of 4 ppm.

Generally, the p*K*_a_ values in the isolated thumb were elevated (∼1 pH unit) compared to those expected for acidic amino acids in globular or disordered proteins. This shift likely reflects the stacking of these along the helices, resulting in proton sensitivity extending into a range relevant to both physiological and pathological conditions (pH 5.5–7.6). Notably, this elevation is observed even for residues not directly facing the palm in the intact full‐length channel, underscoring that the proton sensitivity of the thumb is an intrinsic property, rather than solely dictated by interdomain interactions. Positive cooperativity was observed for H328 (Hill slope of 2.2) and to a lesser extent in the glutamic acid residues, reflecting increased proton sensitivity with decreased pH. Finally, the change in chemical shift in D350 is unusually small, with the protonated state chemical shift similar to the deprotonated state. This residue also displays strong negative cooperativity, providing experimental evidence for a potential proton wire forming, involving D350 and its neighbors, E354, and D346. E314, D332, D354, and D356 were not included in the analysis as they could not be detected at neutral pH. To further investigate the role of these acidic residues in pH‐dependent channel gating, we performed electrophysiology experiments with alanine mutants of 16 protonatable residues in full‐length cASIC1a. We determined the pH‐dependence of activation and steady‐state desensitization (SSD) for each channel (Figure 6f, g, S17, and Table S8) and compared these functional shifts with the residue‐specific p*K*_a_ values measured for the isolated thumb domain. In general, we find the strongest effect on pH related parameters to be due to mutations of aspartic acid residues and the histidine residue. This correlates with the exchange broadening seen most strongly in the aspartic acid residues during the NMR chemical shift titrations. Notably, D346 and D356 mutations had the most disruptive effects on proton sensitivity (see red background shading in Figure 6e, f). In strong agreement, D346 showed the highest estimated p*K*_a_ of any aspartic acid residues, ~1.5 pH units above the expected range for aspartic acids. D356 could not be detected by NMR, suggesting conformational dynamics in this residue even at neutral and basic pH. Mutations in D350, E358, and H328 were significantly but slightly less disruptive to pH sensing. E358 shows one of the highest p*K*_a_ values of the glutamic acid residues measured, while D350, as noted, shows an unusual profile with strong negative cooperativity, possibly due to sharing of protons with neighboring acids (D346 and E354; see Figure 6a, c).

While mutation of individual residues did not drastically alter pH dependence in ASIC1a, our NMR data reveals an order of protonation events within the isolated thumb domain. Each residue undergoes protonation at distinct stages of acidification, depending on its local environment. This cumulative effect of multiple protonation events reinforces that no single protonation event dictates ASIC gating transitions, but rather that the combined influence of these events contributes to the channel's pH sensitivity and rationalizes the observed redundancy in proton sensing from previous mutagenesis studies [7, 10, 11, 12, 28, 31, 32], and may explain the variability of acidic residue conservation across ASIC subtypes [1].

The elevated p*K*_a_ values of the acidic residues on the thumb domain are also relevant to Ca^2+^ coordination within the acidic pocket of ASICs, which includes thumb‐domain residues on helix‐5 [33, 34]. In contrast to H^+^ and PcTx1, Ca^2+^ binding is thought to stabilize the resting state of the channel by competing with H^+^. To explore the Ca^2+^ binding interface of the isolated thumb domain, we performed additional ^15^N HSQC chemical shift mapping experiments (Figure S18). Ca^2+^ addition results predominantly in fast exchange, with chemical shift changes that are relatively small and spatially localized, consistent with weak, but specific binding. Perturbations cluster on helix‐5, where residues E339, Y341, K342, A345, D346, D350, F351, and D356 show the largest chemical shift changes, while residues L349, F351, V353, D356, and E354 signals are also broadened upon Ca^2+^ addition. Notably, the helix‐4–helix‐5 loop region, which exhibits strong chemical shift perturbations in our pH titration series, shows comparatively weak Ca^2+^ sensitivity, with V327 showing a significant chemical shift change while both V327 and H328 show signal broadening (intermediate exchange). Two acidic residues in helix‐5, D346 and D350 exhibit slow‐exchange behavior upon Ca^2+^ addition, consistent with stronger and specific Ca^2+^ interactions. Interestingly, both residues show unusual p*K*_a_ curves where D346 appears to have the most elevated p*K*_a_ of any aspartic acid residue measured, and D350 shows evidence of negative cooperativity. The NMR data suggest that Ca^2+^ primarily competes with H^+^ binding at this region of thumb, centred on D346 and D350.

Taken together, our data delineate distinct modes of modulation encoded within the isolated thumb domain. Protonation and PcTx1 binding both induce widespread chemical shift perturbations and exchange broadening (Figures S9 and S14), consistent with conformational rearrangements associated with gating transitions. In contrast, Ca^2+^ binding produces comparatively localized perturbations centred on helix‐5 residues, without evidence of a comparable conformational shift (Figure S18), consistent with its role in stabilizing the resting state at neutral pH. Similarly, DynA 2–17 binds to the thumb without eliciting large‐scale conformational rearrangements or functional modulation (Figure S3), demonstrating that ligand binding alone is not sufficient to drive gating‐relevant transitions. These findings demonstrate that, the isolated thumb domain not only preserves the structural determinants of ligand binding, but also captures key elements of the conformational landscape underlying ASIC gating. As such, the thumb constitutes a minimal yet functionally informative module through which proton‐, peptide‐, and metal‐dependent modulation can be dissected in solution. Future structural and kinetic studies exploiting this tractable system will enable a more precise understanding of how local chemical events are coupled to global channel transitions in ASICs.

Finally, it is important to note while the isolated thumb domain reproduces key features of ligand recognition and proton‐dependent conformational behavior, it does not fully capture the structural environment of the intact channel. In particular, interactions involving neighboring extracellular domains and the complementary face are absent in the isolated system and likely contribute to the higher binding affinities and full functional modulation observed in the intact channel. Consequently, the isolated thumb should be viewed as a reductionist platform that enables detailed biophysical interrogation of local interactions and dynamics, while complementary studies in the full‐length channel remain essential for understanding the complete mechanism of ASIC gating.

## 3 Conclusions

This study establishes the isolated ASIC1a thumb domain as a versatile model for investigating ASIC ligand interactions, pH sensitivity, and channel gating. While the system has limitations, particularly in its application to multi‐domain ligands and the absence of neighboring non‐thumb residues, and therefore cannot fully recapitulate all aspects of channel pharmacology, its small size enables biophysical measurements that are difficult to obtain in the intact membrane protein. The ability to characterize thumb domain interactions across ASIC subtypes and to explore both endogenous and exogenous modulators underscore its value for basic research and future therapeutic development. In this context, the thumb domain provides a tractable platform for screening and structure‐guided studies of ASIC ligands. We speculate that future studies using this system could uncover novel therapeutic leads for treatment of conditions related to ASIC dysfunction, such as pain, ischemic stroke, and neurodegenerative diseases.

## Conflicts of Interest

The authors declare no conflicts of interest.

## Supporting information




**Supporting File 1**: The authors have cited additional references within the Supporting Information [35, 36, 37, 38, 39, 40, 41, 42, 43, 44, 45, 46].

**Supporting File 2**: anie71882‐sup‐0002‐Data.zip.

## Data Availability

The data that support the findings of this study are available from the corresponding author upon reasonable request.
