# Supplementary material for: The Isolated Thumb Domain of Acid‐Sensing Ion Channels Forms a Minimal Folding Unit Enabling Ligand Binding Studies
Source: Angew Chem Int Ed Engl. 2026 Mar 25;65(19):e23977. doi: 10.1002/anie.202523977 (PMC13134594; doi:10.1002/anie.202523977)
Supplement: Supplementary file 1 — Supporting File 1: The authors have cited additional references within the Supporting Information [35, 36, 37, 38, 39, 40, 41, 42, 43, 44, 45, 46]. [file ANIE-65-e23977-s002.pdf]

Supporting Information

The Isolated Thumb Domain of Acid-Sensing Ion Channels Forms a Minimal Folding Unit Enabling Ligand Binding Studies

Biswa P. Mishra,[a] Ben Cristofori-Armstrong,[a,b] Elena Budusan,[b] Mimi Golder,[a] Neville J. Butcher,[b] Junyu Liu,[a] Theo Crawford,[a] Yanni K.-Y. Chin,[a] Anneka Pereira Schmidt,[a] Xinying Jia,[a] Taylor B. Smallwood,[b] Richard J. Clark,[b] Jan P. Wurm,[c] Lachlan D. Rash,*[b] and Mehdi Mobli,*[a]

[a] Dr. B. P. Mishra, Dr. B. Cristofori-Armstrong, M. Golder, Dr. J. Liu, Dr. T. Crawford, Dr. Y. K.-Y. Chin, A. P. Schmidt, Dr. X. Jia, Prof. M. Mobli
Australian Institute for Bioengineering and Nanotechnology
The University of Queensland
St Lucia, QLD 4072, Australia
E-mail: m.mobli@uq.edu.au

[b] Dr. E. Budusan, Dr. N. J. Butcher, Dr. T. B. Smallwood, Dr. R. J. Clark, Assoc. Prof. L. D. Rash
School of Biomedical Sciences
The University of Queensland
St Lucia, QLD 4072, Australia
E-mail: l.rash@uq.edu.au

[c] Dr. J. P. Wurm
Bruker Biospin
Rudolf-Plank-Straße 23, 76275 Ettlingen, Germany

Experimental Methods

Peptide production

Recombinant ASIC thumb, PcTx1, and mutants were produced using previously described expression methods.[1] Briefly, codon optimised gene constructs for each peptide were synthesised and subcloned into a His6-maltose binding protein-tobacco etch virus (TEV)-peptide vector. Fusion proteins were expressed in *E. coli* (DE3) cells at 37 °C till an OD_{600} of ~1 was reached and induced with 0.5 mM isopropylthiogalactopyranoside overnight at 16°C. When producing $^{15}N/^{13}C$-labelled peptides, cells were induced in minimal media containing $^{15}NH_4Cl$ and ^{13}C glucose as the sole nitrogen and carbon source. Cells were collected by centrifugation and disrupted in buffer containing 40 mM Tris, 400 mM NaCl, pH 8. Cell extracts were centrifuged at 40000 *g* for 30 minutes and the fusion protein captured from the soluble fraction using Ni-nitrilotriacetic acid (Ni-NTA) beads. After elution from the beads, fusion tags were removed by TEV cleavage overnight at room temperature, followed by purification using reversed-phase high-performance liquid chromatography (RP-HPLC)(Phenomenex Jupiter 300Å, 10 µm, 10 x 250 mm). The correct product was then confirmed by matrix-assisted laser desorption/ionisation time-of-flight (MALDI-TOF) mass spectrometry. Mutagenesis of PcTx1 and the cASIC1a thumb domain was performed using standard PCR-based site-directed mutagenesis, and all constructs were sequence-verified by Sanger sequencing before use.

Synthetic BigDyn was synthesised on a CS Bio Co. CS136X peptide synthesiser using Fmoc (9-fluorenylmethyloxycarbonyl)-based solid phase peptide synthesis (SPPS) on a low loaded 2-chlorotrityl chloride resin 100-200 mesh (0.2 mmol/g; Mimotopes Pty Ltd.) at a 0.125 millimole scale. 20% piperidine (Merck, Germany) was used to remove Fmoc for elongation of the peptide chain (2 x 5 minutes). A ratio of 1:4:4 molar equivalents of AA:HBTU:DIPEA were used for each amino acid coupling. All isoleucine and arginine residues were double coupled. Following chain assembly, the peptide was cleaved from the resin and side chain protecting groups removed by treatment with TFA/TIPS/H_2O/DODT at a ratio of 92.5:2.5:2.5:2.5 for 2 hours at 23 °C. The crude peptide was then precipitated with diethyl ether (Merck, Germany), filtered and re-solubilised in 50/50 buffer A/B (A = water with 0.5% trifluoroacetic acid, B = 90% acetonitrile and 10% water with 0.045% trifluoroacetic acid) before being lyophilised. The crude peptide was purified using RP-HPLC with an increasing gradient of 1% buffer B in buffer A per minute over 80 minutes (Phenomenex Jupiter 300Å, 10 µm, 21.2 x 250 mm). HPLC fractions were analysed by ESI-MS and fractions containing the desired product were combined and lyophilised. Synthetic DynA 2–17 was obtained commercially (Thermo Fisher Scientific, US).

Pulldown assay

rASIC1a thumb was dissolved in 100 mM sodium phosphate buffer pH 8, mixed with 5x molar equivalents of sulfo-NHS-Biotin (Thermo Fisher Scientific) and incubated at 4 °C for 16 hours. Biotinylated products were purified by RP-HPLC (Agilent ZORBAX 300SB-C3 5 µm, 9.4 x 250 mm), fractions containing one or two biotin additions were pooled and lyophilised. Biotinylated rASIC1a thumb was dissolved in 1.5 mL PBS at a

concentration of 15 µM and mixed with 1 mL avidin-agarose resin pre-equilibrated with PBS. Unbound rASIC1a thumb was removed using a spin column and the avidin-resin washed twice with 1 mL PBS. A separate control avidin-resin column was prepared in the same manner without the addition of rASIC1a thumb. The peptide mixture used for the pulldown assay contained PcTx1 (10 µM), BigDyn (10 µM), and an irrelevant control peptide, soricidin (10 µM; UniProt ID: P0C2P6), which was included to assess non-specific binding. Soricidin was enzymatically digested prior to the assay, resulting in multiple peptide fragments and corresponding HPLC peaks. Additional peaks in the chromatograms arise from singly and doubly biotinylated rASIC1a thumb species. The peptide solution was applied to the rASIC1a thumb and control avidin-resin columns, unbound peptide was removed by centrifugation and the resin washed twice with 1.5 mL PBS. Bound peptides were then removed using 0.1 M glycine pH 2.8. All samples were analysed using RP-HPLC (Agilent ZORBAX 300SB-C18 5 µm, 4.6 x 250 mm).

NMR structure determination

The NMR sample of $^{15}N/^{13}C$ labelled cASIC1a thumb contained 50 mM Bis-Tris buffer (pH 7.0) with 1 mM EDTA and 3 mM NaN_3 in a 95/5% (v/v) H_2O/D_2O mixture. All the following spectra were recorded with sample concentration of 315 µM at 25 °C in a volume of 350 µL taken in a susceptibility-matched 5 mm microtube (Shigemi Inc. Japan), on a Bruker Neo NMR spectrometer operating at 900 MHz resonance frequency, equipped with a triple resonance cryoprobe. 3D experiments (excluding NOESY experiments) were acquired by non-uniform sampling (NUS) and reconstructed using maximum entropy reconstruction.[2] Sampling schedules were designed to follow the decay profile of the indirect dimensions. Decay rates that are equivalent to 15 Hz line width were used for non-constant time (non-CT) dimensions whereas no decay was used for CT dimensions. Sched3d software was used to generate all sampling schedules.[3]

2D (^{15}N-^{1}H) HSQC, 2D (^{13}C-^{1}H) HSQC, 3D, 3D CBCA(CO)NH, 3D HNCACB, 3D HBHA(CO)NH, 3D C(CO)NH, 3D H(CO)NH, 3D ^{13}C-edited NOESY (for aliphatic and aromatic regions) and ^{15}N-edited NOESY spectra were recorded and used for resonance assignment and extraction of NOE restraints for structure calculation (details in Table S1).

Maximum-entropy method was used to reconstruct all non-uniformly sampled spectra. All spectra were processed using the Rowland NMR toolkit[4] and analysed using CCPNMR program.[5] All NOESY cross-peaks were manually picked and supplemented with dihedral angles derived from chemical shifts using the TALOS software.[6] These angles were used for structure calculations using CYANA v3.98.13,[7] which automatically assigned all the NOEs. A total of 15000 structures were calculated and an ensemble of 20 structures with the lowest CYANA target function were selected.

NMR chemical shift mapping

^{1}H-^{15}N HSQC spectra were acquired on a Bruker Avance Neo NMR spectrometer operating at 900 MHz resonance frequency equipped with a triple resonance cryoprobe. All ligand titrations (PcTx1, BigDyn, DynA 2-17, Ca^{2+}) were performed

using samples dissolved in 50 mM Bis-Tris buffer (pH 7.0) with 1 mM EDTA (omitted in the Ca^{2+} titration) and 3 mM NaN_3 in a 95/5% (v/v) H_2O/D_2O mixture and recorded at room temperature. All ^1H-^{15}N HSQC titrations were carried out under identical experimental conditions of 4 scans and 128 complex points in the indirect dimension. Two sets of PcTx1-cASIC1a thumb titrations were performed – one where cASIC1a thumb was labelled with ^{15}N, and another with ^{15}N labelled PcTx1. Titrations between DynA 2–17 and ^{15}N-cASIC1a thumb were performed under identical experimental conditions of 4 scans and 128 complex points in the indirect dimension. Each individual ^1H-^{15}N HSQC spectrum of cASIC1a thumb (acquired with and without ligand) was processed by Topspin 4.0.7 (Bruker, Massachusetts, USA) and the Rowland NMR toolkit (University of Connecticut, USA). Peak assignments were taken from the solved structure of cASIC1a thumb. Each individual ^1H-^{15}N HSQC spectrum of PcTx1 (acquired with and without cASIC1a thumb) was also processed in the same way. Peak assignments were performed in CCPNMR,[5] by using previously reported ^{15}N-^1H chemical shifts for PcTx1 (BMRB entry 16468, PDB ID 2KNI).[8] The chemical shift changes ($\Delta\delta$) were calculated (Equation 1, $\alpha = 0.14$) and plotted to characterise the residues undergoing significant perturbations upon the addition of different ligands.

$$\Delta\delta=\sqrt{\frac{1}{2}\left[\delta_H^2+\left(\alpha\ \delta_N^2\right)\right]} \qquad (1)$$

For pH titration a $^{15}N/^{13}C$ labelled cASIC1a thumb sample was used in 100 mM KCl, 2 mM Tris, 2 mM formate, 2 mM piperazine, 2 mM imidazole for internal pH monitoring and 10 µM DSS for referencing, and 5% D_2O. pH of the NMR samples was adjusted by addition of small increments of sterile stocks of 0.1 M HCl or NaOH. The spectra were recorded with a sample concentration of 180 µM at 25 °C in a volume of 350 µL taken in a susceptibility-matched 5 mm microtube (Shigemi Inc. Japan), on a Bruker Avance NMR spectrometer operating at 900 MHz resonance frequency equipped with a triple resonance cryoprobe with an interscan delay of 1.0 s. Each individual ^1H-^{15}N HSQC spectrum of cASIC1a thumb was processed as described above, and the chemical shift changes were calculated and plotted to characterise the residues undergoing maximum perturbations with drop in pH.

CPMG relaxation dispersion analysis

^{15}N CPMG relaxation dispersion experiments were recorded using a 100 µM sample of ^{15}N-labelled cASIC1a thumb in a buffer consisting of 100 mM KCl, 2 mM Tris, 2 mM formate, 2 mM piperazine, 2 mM imidazole for internal pH monitoring and 10 µM DSS for referencing, and 5% D_2O. The pH was adjusted to 6 and confirmed using the internal standards. The NMR data were acquired on a Bruker Neo spectrometer equipped with a cryogenically cooled triple resonance probe, operating at nominal ^1H frequency of 900 MHz. The experiment used a constant total CPMG relaxation period (T = 40 ms), with effective CPMG field strengths (ν_{CPMG}) varied by changing the refocusing pulse repetition rate (ν_{CPMG} = 0, 50x2, 100, 150, 200, 250, 350, 500, 750, 1000 s^{-1}). The data were acquired as a pseudo-3D experiment with temperature compensation and scrambling of the ν_{CPMG} values.[9] 70 complex data points (real +

imaginary) were acquired along the ^{15}N dimension with signal averaging of 64 scans per increment and an interscan delay of 2 s. The data was extended (doubled) by linear prediction using the Rowland NMR toolkit, which was also used to extract peak intensities.[4] Peak intensities were extracted directly from the spectra using box integration centred on each resonance (sum 3x3 digital points). Effective transverse relaxation rates ($R_{2,eff}$) were calculated as:

$$R_{2,eff}(\nu) = -\frac{1}{T} ln\left(\frac{I(\nu)}{I_0}\right) \quad (2)$$

where I_0 corresponds to the reference intensity at $\nu = 0$. For residues measured at replicate ν values, intensities and $R_{2,eff}$ values were averaged. Uncertainties in $R_{2,eff}$ were estimated by combining propagated spectral noise with empirical measurement variability derived from replicate ν_{CPMG} points; these uncertainties are shown as error bars in the dispersion profiles.

NMR measurements for pK_a determination of protonatable acidic and histidine residues of cASIC1a thumb

The sample used in the pH titration (in multinidicator buffer) was also used for the measurement of pK_a values. For His pK_a measurements, 2D high-resolution (^1H -^{13}C) HSQC spectra were recorded from pH 8.3 to 2.5 (11 pH points) for assigning the $^{13}C_ε$ resonances. The time domain matrix consisted of 64 (t1, ^{13}C) x 2048 (t2, ^1H) complex points with acquisition times of 2.8 ms (t1), and 69.6 ms (t2) using 32 scans per FID with the ^{13}C carrier at 125 ppm.

For Asp and Glu pK_a measurements, 3D HCACO spectra were recorded from pH 8.3 to 2.5 (11 pH points) for assigning the $^{13}C_γ$ (in Asp) and $^{13}C_δ$ (in Glu) resonances. The time domain matrix consisted of 160 (t1, ^{13}C) x 200 (t2, ^{13}C) x 2048 (t3, ^1H) complex points with acquisition times of 2.7 ms (t1), 14.7 ms (t2) and 69.6 ms (t3) using 80 scans per FID with the ^{13}C carrier at 40 ppm. The spectra were acquired using non-uniform sampling (NUS) with 200 data coordinates. Maximum-entropy method was used to reconstruct all non-uniformly sampled spectra. All NMR spectra were processed using the Rowland NMR toolkit and analysed using CCPNMR.

NMR measurements for pK_a determination of basic residues in PcTx1

The NMR sample of ^{15}N/^{13}C labelled PcTx1 contains 100 mM KCl, 2 mM Tris, 2 mM formate, 2 mM piperazine, 2 mM imidazole, 10 μM DSS, and 5% D_2O. pH of the NMR samples was adjusted by addition of small increments of sterile stocks of 0.1 M HCl or NaOH. All the following spectra were recorded with sample concentration of 225 μM at 25°C in a volume of 350 μL taken in a susceptibility-matched 5 mm microtube (Shigemi Inc. Japan), on a Bruker Avance NMR spectrometer operating at 900 MHz resonance frequency equipped with a triple resonance cryoprobe with an interscan delay of 1.0 s.

For Arg and Lys pK_a measurements, 2D high-resolution band-selective (^1H -^{13}C) HSQC spectra with selective decoupling were recorded from pH 5.5 to 12 (9 pH points) for following the $^{13}C_δ$ (in Arg) and $^{13}C_ε$ (in Lys) resonances. The resonances were assigned based on reported assignments at the same pH (BMRB ID 16468). The time domain matrix consisted of 256 (t1, ^{13}C) x 2048 (t2, ^1H) complex points with

acquisition times of 40.4 ms (t1), and 94.2 ms (t2) using 16 scans per FID with the ^{13}C carrier at 39 ppm.

NMR data fitting and pK_a determination

The pH profiles of chemical shifts measured were fitted to the Henderson-Hasselbalch equation (Equation 3) and the best-fit pK_a values were taken.

$$\delta_{obs} = \delta_B + \frac{\Delta\delta \times 10^{n(pK_a - pH)}}{1+10^{n(pK_a-pH)}} \quad (3)$$

where δ_{obs} is the observed chemical shift at a given pH, δ_B is the deprotonated chemical shift, and n is the Hill coefficient. The chemical shift data were fitted and plotted using R (version 4.1.0).

Some residues had very weak signal at pH 5.5 and below, so their resonances couldn't be assigned. The $^{13}C_\gamma$ (in Asp) and $^{13}C_\delta$ (in Glu) usually shift ~4 ppm upfield after getting protonated. For residues where the complete curve could not be constructed, the chemical shift value was fixed at 4 ppm upfield of the first titration point. Then the titration curve was fitted to the Henderson-Hasselbalch to estimate the best-fit pK_a value.

Isothermal titration calorimetry

Binding affinities were studied using a MicroCal iTC200 instrument (Malvern, UK). The thermodynamic parameters of the binding interaction were calculated using MicroCal origin software. These parameters include amount of heat released, the stoichiometry of binding (N), binding enthalpy (ΔH), and the dissociation constant (K_D) in a binding model with a single interaction site. PcTx1 ITC experiments were performed using a cASIC1a thumb (WT and mutants) concentration of 10 μM (in cell) and a peptide (WT and mutants) concentration of 100 μM (in syringe). In dynorphin ITC experiments, the cASIC1a thumb and peptide (BigDyn and DynA 2-17) concentrations were 50 μM (in cell) and 500 μM (in syringe) respectively. All ITC experiments were performed in a 50 mM Citrate-Phosphate buffer at pH 7. The peptides (thumb and ligand) were double lyophilised (from HCl) to remove traces of TFA.

For measurement of proton transfer effects PcTx1 (100 μM) was titrated against 10 μM of cASIC1a thumb in 13x3.2 μL injections at 25°C. Two independent experimental batches were used, each in either 50 mM HEPES (pH 7.0) or 50 mM PIPES (pH 7.0) buffer. The ITC experiments for both batches were done in duplicates (n=2), along with a blank experiment where the buffer was titrated against cASIC1a thumb. Data analysis and fitting was performed on MicroCal PEAQ-ITC Analysis software v1.4.1 (Malvern, UK).

The observed enthalpy change (ΔH_{obs}) was analysed using the relationship:

$$\Delta H_{obs} = \Delta H_{bind} + n\Delta H_{ion} \quad (4)$$

where ΔH_{bind} represents the intrinsic, buffer-independent binding enthalpy, ΔH_{ion} is the enthalpy of ionisation of the buffer at 25 °C, and n is the number of protons linked to complex formation. Literature values for ΔH_{ion} were used (HEPES: 20.4 kJ mol^{-1}; PIPES: 11.2 kJ mol^{-1}). The intrinsic binding enthalpy (ΔH_{bind}) and proton linkage parameter (n) were obtained from linear analysis of ΔH_{obs} as a function of ΔH_{ion}.

Xenopus laevis oocyte electrophysiology

Two-electrode voltage clamp experiments were performed with *X. laevis* oocytes expressing chicken and rat ASIC1a.[10] Stage V/VI oocytes were injected with 1–5 ng synthesised cRNA, then incubated at 17°C for 1–3 days in 50% Leibovitz's L-15 medium (Gibco), supplemented with 25 μg/mL gentamicin, 25 μg/mL streptomycin, and 2.5% foetal horse serum. Two-electrode voltage clamp (Axoclamp 900A amplifier; Axon Instruments) were performed at room temperature in ND96 solution (in mM: 96 NaCl, 2 KCl, 1 $MgCl_2$, 1.8 $CaCl_2$, 5 HEPES; pH 7.4 with NaOH) with a ~40 μL recording chamber (gravity-fed perfusion with a flow rate of ~2mL/min). HEPES was replaced by MES to buffer solutions at pH<6.8. In experiments where PcTx1 or mutants was added, solutions were supplemented with 0.05% fatty-acid free bovine serum albumin. Borosilicate glass microelectrodes had resistances of 0.2–0.8 MΩ when backfilled with 3 M KCl. Data were digitised at 5 kHz and filtered at 0.1 kHz using pCLAMP 11 software (Digidata 1550B; Axon Instruments).

The pH application conditions for BigDyn and DynA 2–17 experiments are described in the figure and legend. PcTx1 and mutants were assayed by applying the peptide at the pH noted in figures, and the direct effect of peptide application or the effect on the subsequent low pH stimulus analysed. Activation curves were determined by conditioning oocytes at pH 7.6 for 55 s and applying a stimulus pH between 7.2 to 4.5 for 5 s. Steady-state desensitisation (SSD) was determined by applying various conditioning solutions of pH values from 7.7 to 6.9 for 115 s prior to stimulation by a pH drop to 5.0 for 5 s.

Data were analysed in GraphPad Prism 10.1.2. All data normalisation is performed as indicated in the y-axis of each electrophysiology figure. Concentration-response and pH-response curves were fitted with a four-parameter logistic (Hill) equation to obtain pEC_{50}, pIC_{50}, or pH_{50} values, and the Hill slope. For the PcTx1 concentration-response data, after curve fitting these values were converted to EC_{50} or IC_{50} and presented in molar (e.g. nM) for better readability. pH_{50} values are kept in logarithmic units as per convention. Data are mean ± standard error of the mean (SEM), and the number of replicates (n) represent independent oocytes. Differences in pH_{50} (ΔpH_{50}) were calculated relative to wild type and the associated uncertainty obtained by propagation of standard errors assuming independent measurements. Statistical tests are specified in the corresponding figure legends. A P value < 0.05 was considered statistically significant in all tests.

X. laevis were purchased from NASCO (Fort Atkinson, WI, USA). *X. laevis* oocyte surgeries were reviewed and approved by the Anatomical Biosciences group of the Animal Ethics Committee at The University of Queensland (QBI/AIBN/087/16/NHMRC/ARC and SBMS/097/19) and conducted in accordance with Australian quarantine regulations.

Supporting Figures

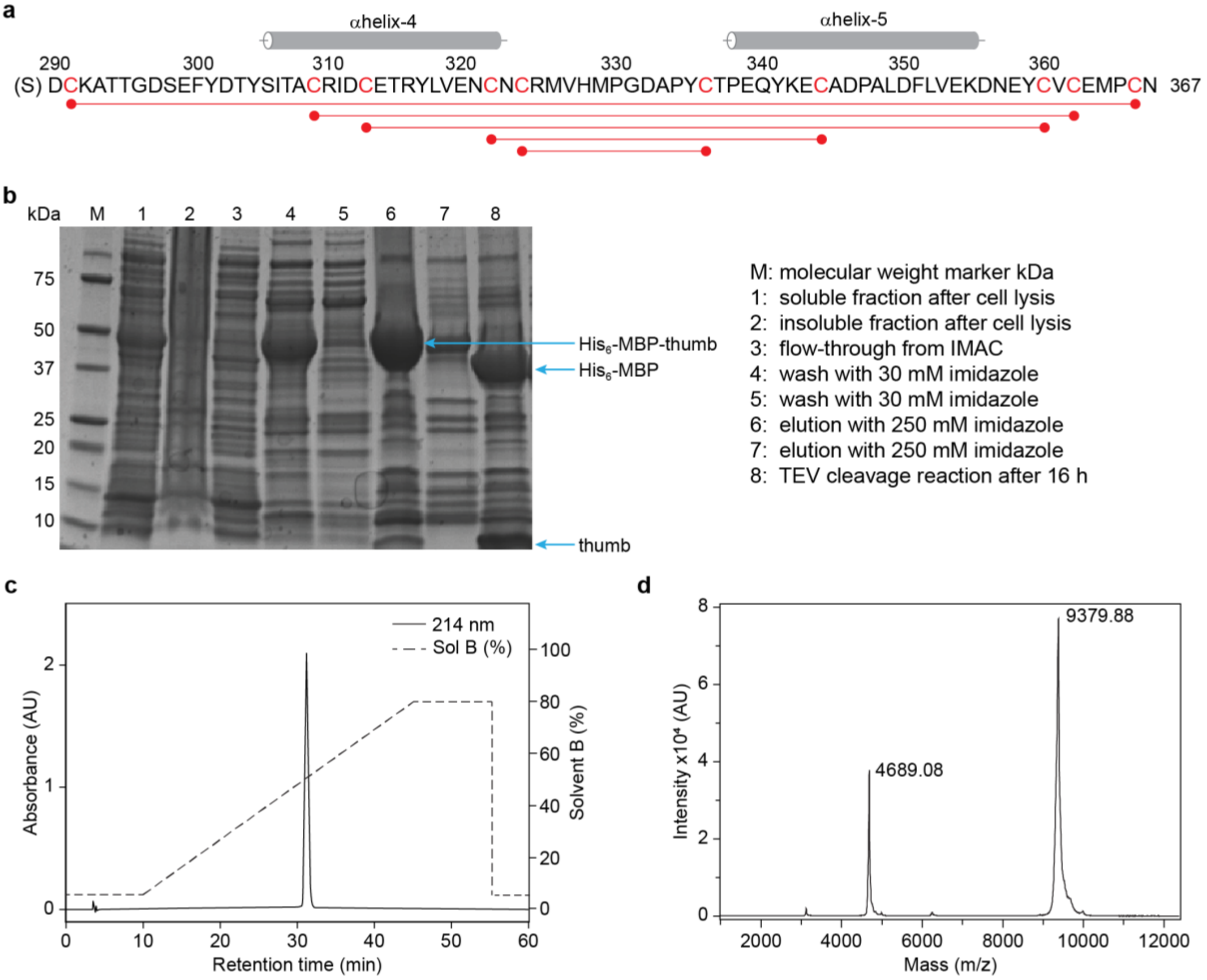

Figure S1: Recombinant production of the cASIC1a thumb domain. a Amino acid sequence of cASIC1a thumb domain with disulfide connectivity indicated. **b** Coomassie-stained SDS-PAGE gel of different steps in the expression and purification protocol; lane M, molecular weight marker in kDa; lane 1, soluble fraction after cell lysis; lane 2, insoluble fraction after cell lysis; lane 3, flow-through after loading of the supernatant onto a Ni^{2+}-NTA beads; lanes 4 & 5, eluate after removing the loosely bound impurities by 4 column volumes of buffer containing 30 mM imidazole; lanes 6 & 7, eluate after passing buffer containing 250 mM imidazole through a Ni^{2+}-NTA column; lane 8, cleavage reaction mixture at 16 hours after addition of TEV protease. **c** RP-HPLC chromatogram after purification of TEV cleaved cASIC1a thumb. The gradient of solvent B (Sol B) is indicated by the dashed line. **d** Matrix-assisted laser desorption/ionization-time of flight mass spectrum (linear mode) of the final purified $^{15}N/^{13}C$ labelled product. The observed mass is in close agreement with the predicted mass of 9377.00 Da.

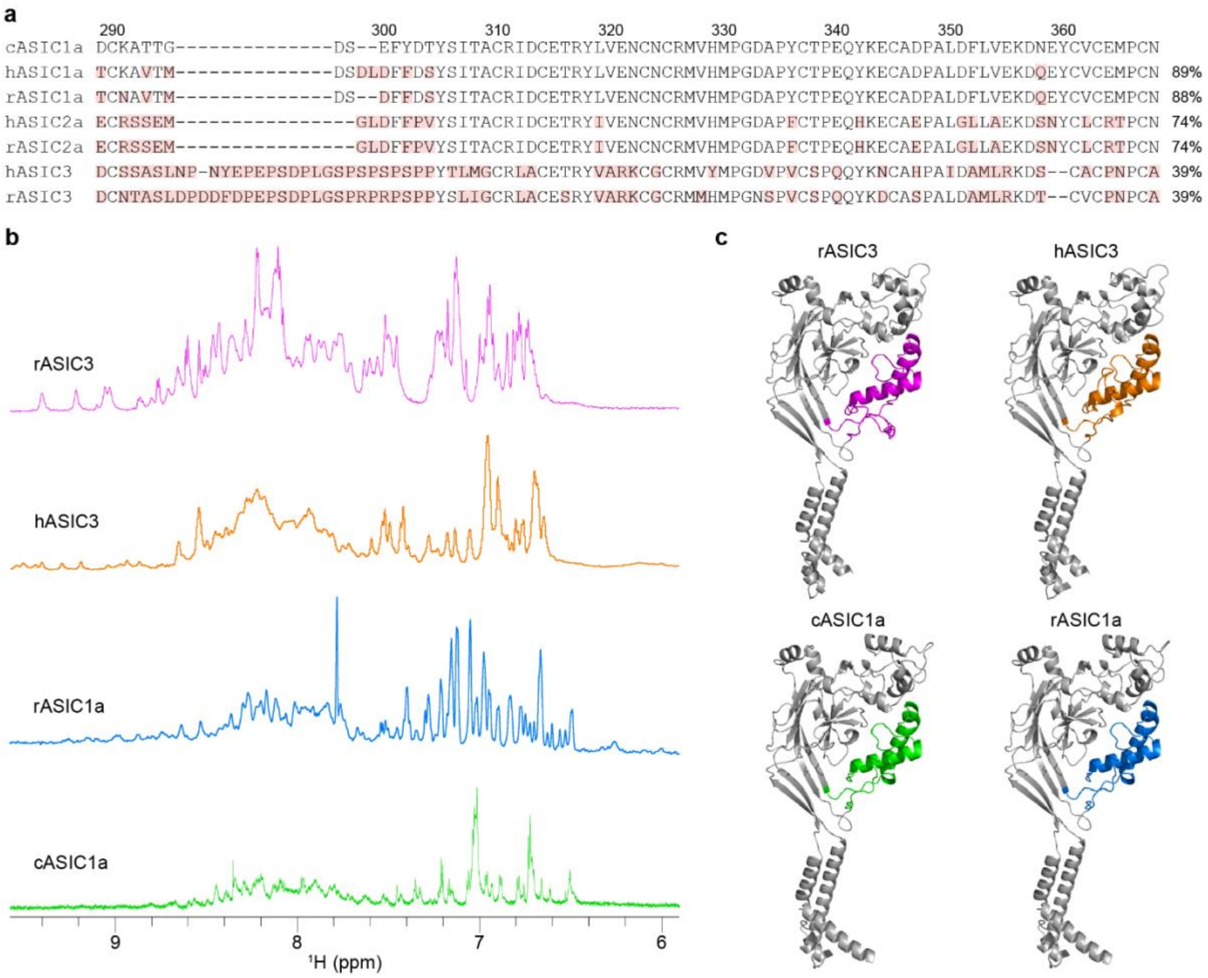

Figure S2: Production of ASIC thumb domains from different species and subtypes. a Sequence alignment of selected ASIC thumb domains, with residues differing from the chicken ASIC1a thumb highlighted in red shading. The percent identity compared to chicken ASIC1a is indicated on the right. **b** ^{1}H-NMR spectra of different thumb domains. The amide region displays well-dispersed signals, indicating that each peptide is in a stable globular fold. **c** AlphaFold 3 models of full-length ASICs from which thumb domains were produced. The thumb domain residues that were produced are highlighted in colours corresponding to the 1D spectra shown in panel b.

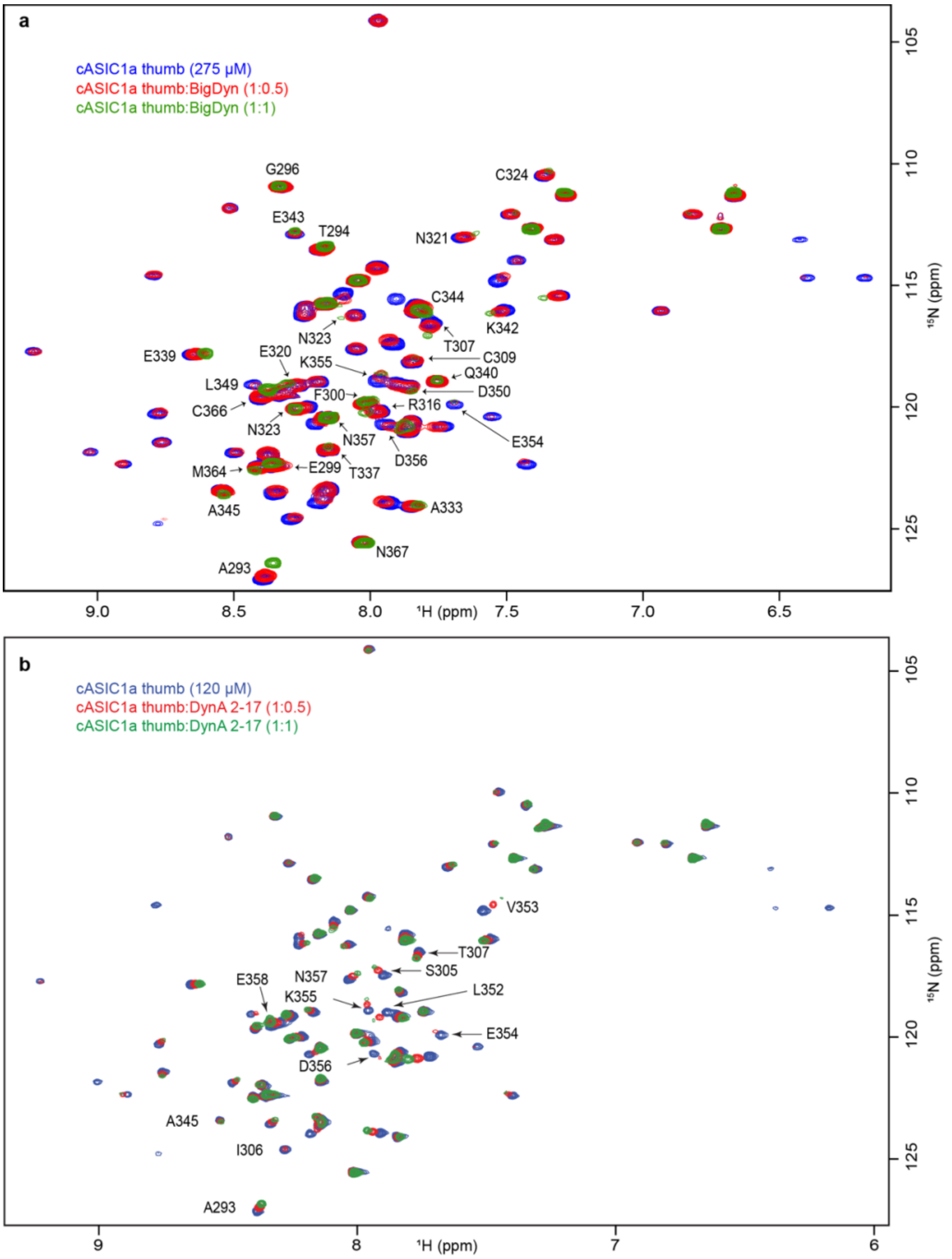

Figure S3: Chemical shift mapping of the ^{15}N-cASIC1a thumb with the addition of Dynorphins. a ^{1}H-^{15}N HSQC spectra of the cASIC1a thumb (275 μM in 50 mM Bis-Tris pH 7.0, 3 mM NaN_3, 1 mM EDTA, 10 μM DSS). Reference spectrum in absence of BigDyn (blue) superimposed with spectra upon addition of BigDyn at a molar ratio of 1:0.5 (red) and 1:1 (green). cASIC1a thumb residues with chemical shift change ($\Delta\delta$) > 0.01 have been labelled. **b** ^{1}H-^{15}N HSQC spectra of the cASIC1a thumb (120 μM in 50 mM Bis-Tris pH 7.0, 3 mM NaN_3, 1 mM EDTA, 10 μM DSS). Reference spectrum in absence of DynA 2-17 (blue) superimposed with spectra upon addition of DynA 2-17 at a molar ratio of 1:0.5 (red) and 1:1 (green). cASIC1a thumb residues in the proposed binding interface labelled.

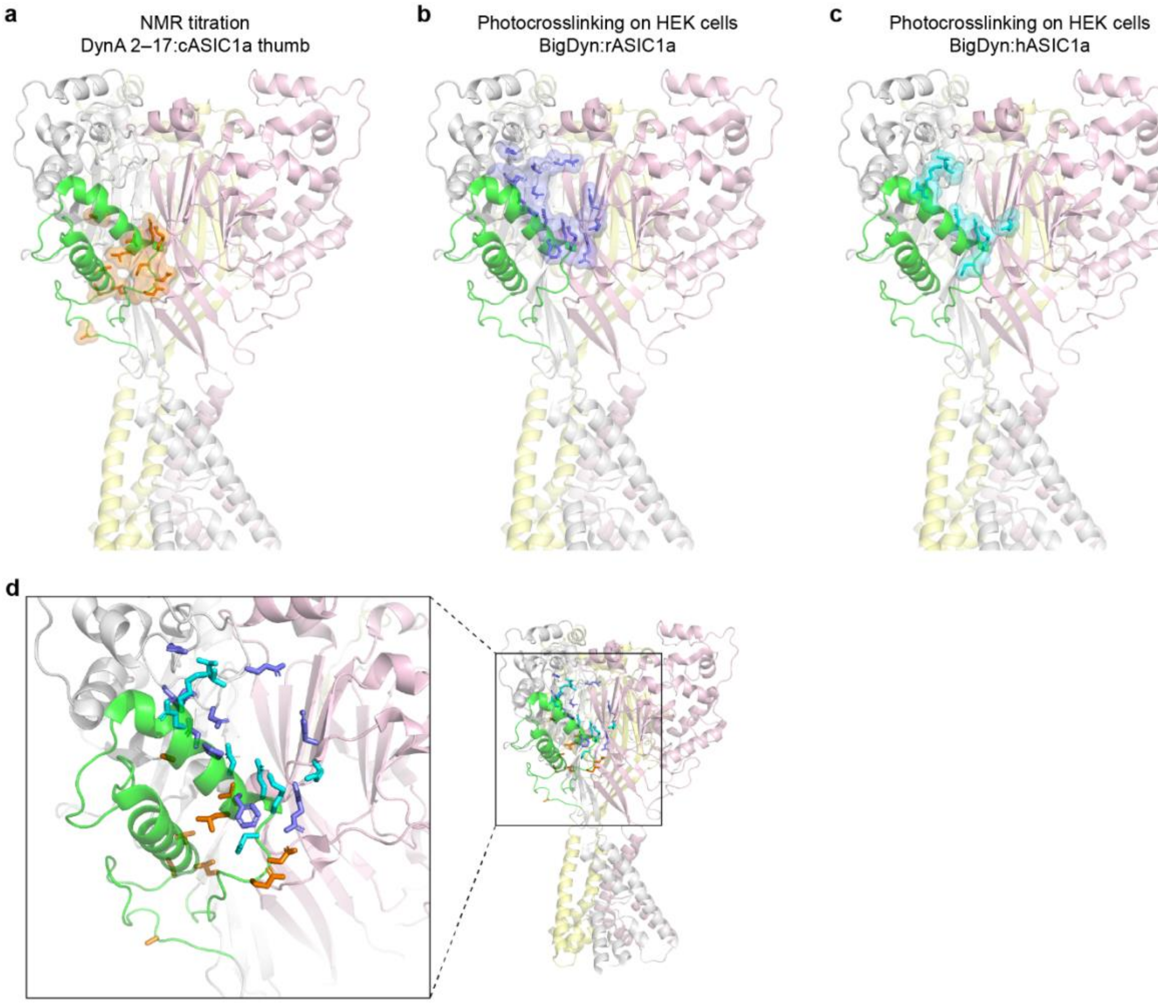

Figure S4: Comparison of dynorphin binding site data at ASIC1a from NMR and crosslinking experiments. a Structure of the cASIC1a trimer (PDB: 5WKU) with one thumb domain highlighted in green. Residues with significant chemical shift changes from NMR titration experiments with DynA 2–17 and the isolated cASIC1a thumb domain are shown in orange as stick and surface representations (see Figure 2e and f). **b** The same cASIC1a trimer as in panel a, with purple side chains indicating residues that showed positive photocrosslinking between BigDyn and ASIC1a when expressed in mammalian cells.[11] **c** A second photocrosslinking study mapping BigDyn interactions with ASIC1a, highlighting interacting residues in cyan.[12] **d** Combined view of all interacting residues identified in both NMR and crosslinking studies. Residues are shown as sticks, colour-coded according to the corresponding panels: orange (NMR, panel a), purple (crosslinking, panel b), and cyan (crosslinking, panel c). Overlap in the lower thumb residues suggests that the N-terminal segment of BigDyn (corresponding to DynA) may bind this region of ASIC1a.

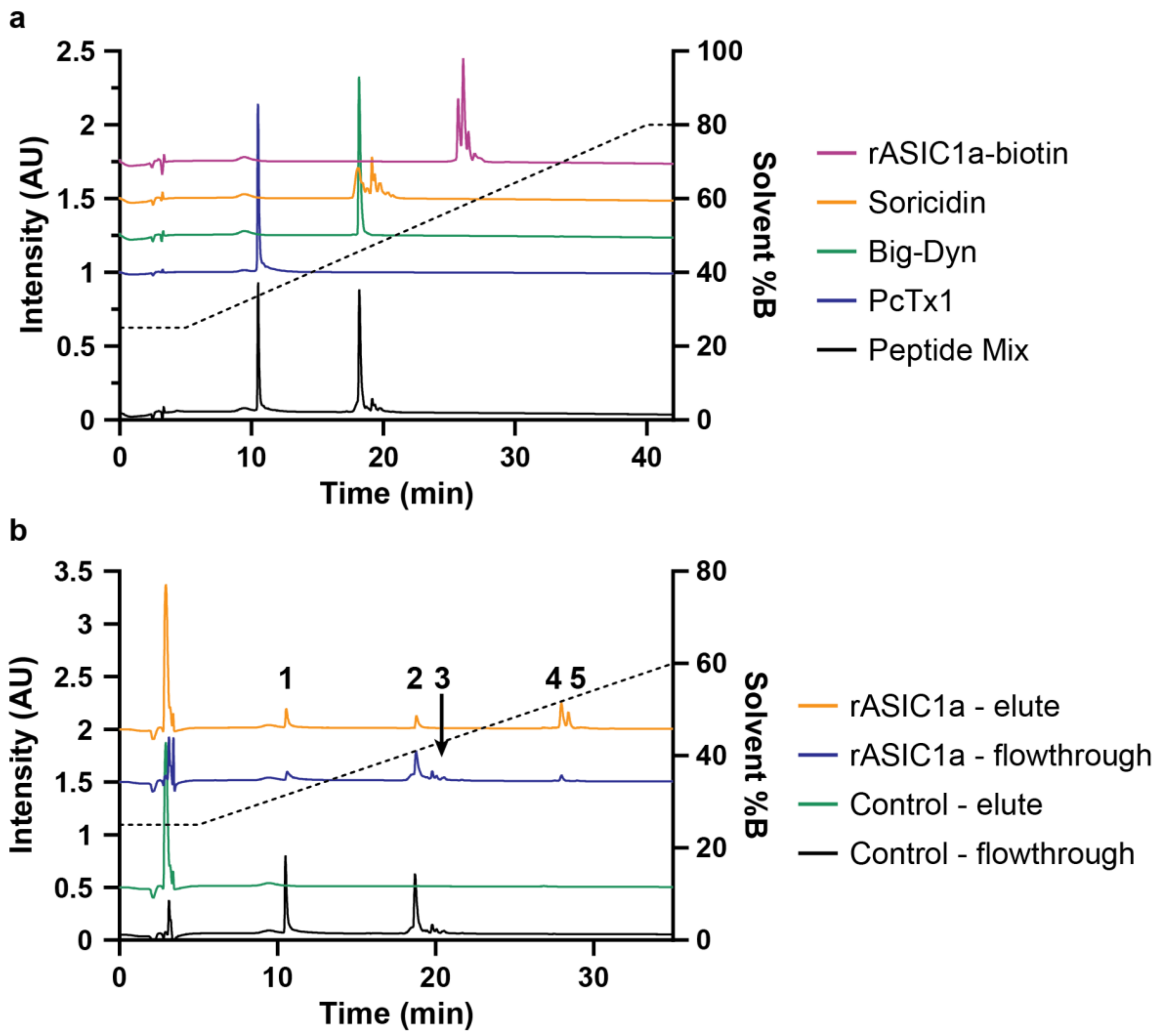

Figure S5: rASIC1a pulldown HPLC chromatograms. a Reverse phase-HPLC chromatograms of biotinylated rat ASIC1a thumb domain (rASIC1-biotin), soricidin, big dynorphin (Big-Dyn), Psalmotoxin-1 (PcTx1), and peptide mixture used for pulldown assay. Enzymatically digested soricidin was included as an irrelevant peptide as a control for non-specfic binding. **b** Reverse phase-HPLC chromatograms for pulldown assay. The peptide mix was applied to rASIC1-biotin avidin resin or control avidin resin, bound (elute) and non-bound fractions (flowthrough) were analysed (1 – PcTx1, 2 – Big-Dyn, 3 – soricidin, 4 – rASIC1a single biotin, 5 – rASIC1a double biotin). Dashed line represents solvent B percentage (right y-axis), HPLC solvent A: 0.05% TFA in H_2O, HPLC solvent B: 0.043% TFA in 90% acetonitrile/H_2O. Chromatograms are offset for visualisation.

```
                                             α helix
ASIC1    DCK---------ATTGDS------EFYDTY--SITACRIDCE-----TRYLVENCNCRMVHMPGD-------
BASIC    WGE---------CNPNIK-----LQNFSSY--STSGCLKECK-----AQHIKKQCGCVPFLLPGY-------
TadNaC1  WGN---------CGQKKL------KYFKSY--SRASCQLECL-----ADSYRRRCDCRTPYMPGS-------
TadNaC4  WGE---------CGSRKL------LFHEFY--SHVACIDEVS-----AIYASTLCNCSIIGAFGP-------
TadNaC8  YGH---------CGSKKL------AFYKKY--TYAGCQISCK-----TEYVQKKCGCRAPDMPGQNV-----
HyNaC2   YATK--------CGSKPL------KNYQIY--RQSSCFLEQL-----GDAIETKCKCKSSFMAGRN------
FaNaC    YGN---------CTEDSL------EGIQTYRNTFFACLQLCK-----QRRLIRECKCKSSALPDLSV-----
ENaCα    YGD---------CTKNGSDVPVENLYPSKY--TQQVCIHSCF-----QESMIKECGCAYIFYPRPQ------
deg-1    YGE---------CVETKKVVDRNYI-YAGYDYHPEGCHRSCF-----QNGLIDDCSCGDPRFPVPE------
mec-4    YGD---------CVPDGKT--SDYI-YSNYEYSVEGCYRSCF-----QQLVLKECRCGDPRFPVPE------
unc-8    WGN---------CSDTFRP--VPYI-Y-NEHYSPEGCHRNCF-----QLKVLEICGCGDPRFPLPSE-----
del-8    KGV---------CTNHI-----------------FGRDAECLVRKWYLSNIIIPFNCTLPYLKSITK-----
ppk9     EARATKQRSCRFNYEAEEM-------MTVPIYSFGLCLSECR-----MFFALRVCGCVPHFYRNRCGYNHIF
ppk11    KGLSLQKRGCYFSTERRL------ILSDVY--NQINCLAECR-----TESILKSCGCIPPKSPIEK------
ppk19    YEMSPKKRRCYFDDEKNPTF-----QNTSLTYNRENCLVVCL-----HLVVWKTCQCSLPAFLPPID-----
ppk28    RSIKKSVRRCLFSDENDL------AYYRTY--SRKNCELECE-----AKLLLRECSCVLYYLPRIDP-----
rpk      AEYHPVRRQCFLSHERSL------RFFKVY--TESNCQLECL-----ANFTLTKCGCVKFSMPRNV------

                                       α helix
ASIC1    ---------APYCTPEQY---KECADPALDFL------VEK-----------------DNEYCVCEMPCN
BASIC    ---------GIECDLQKY---FSCVSPVLDHI------EFKDL------------CTVGTHNSSCPVSCE
TadNaC1  ---------SPICSPEHI---KKCVTKYLG-L------STP-----------------ENFTCNCPNDCR
TadNaC4  ---------YGVCDSVKF---ITCIVPILAKA------RVQ----------------ANENIAACPVACE
TadNaC8  ---------IPVCSPQKM---IECVSPNLEKL------RTI-----------------NDKVCICPIPCH
HyNaC2   ---------IPYCSLRQT---VTCLMPTIYDF------DRK-------------------TNNNCPVDCE
FaNaC    -------ENITFCGVIP--DWKDIRRNVTGEYKMNQTIPTISLACEARVQKQLNNDRSYETDCGCYQPCS
ENaCα    --------NVEYCDYRKHSSWGYCYYKLQVDF------SSD----------------HLGCFTKCRKPCS
deg-1    --------GYRHCSAFNA-TARTCLEKNIGSV------------------GDFHHITQKMDKCVCKQSCE
mec-4    --------NARHCDAADP-IARKCLDARMNDL------------------GGLHGS----FRCRCQQPCR
unc-8    --------EHRHCNAKSK-IDRQCLSNLTSDS------------------GGYHHLH---EQCECRQPCH
del-8    -----LPPTTGVCKPDVI--ADNYLDKIQYVW------------------------NSAEVNEECTPGCN
ppk9     GTQMRNGRRLPVCGLEG----IGCLVKIKREI------I------------SL-KSDKYKINCNCLANCD
ppk11    --------SWLICDLKQ----MQCVIDFDHDE------IIS----------------GEQKNCDCLPPCE
ppk19    --------GVPECGIND----AQCLGNNSDIFTYVKM----------GDQEKYINDSRQGHFCDCPDNCN
ppk28    --------LARVCGPND----NQCTDRVQTEI------ESS---------------LTNLSCENCWPGCF
rpk      --------DMPVCGEDK----IHCYDRAEREL------LVREFKRVKALNAGRENSRSVESACNCMPACT
```

Figure S6: Multiple sequence alignment of thumb domains from the broader degenerin/epithelial sodium channel (DEG/ENaC) family. Residues are aligned to their corresponding positions in the cASIC1a thumb domain. Cysteine residues are highlighted in red, emphasising the highly conserved disulfide bonding patterns across family members. Helices are indicated with grey background shading to illustrate secondary structure elements within the alignment. This highlights the structural conservation of the thumb domain despite minimal sequence conservation in this region.

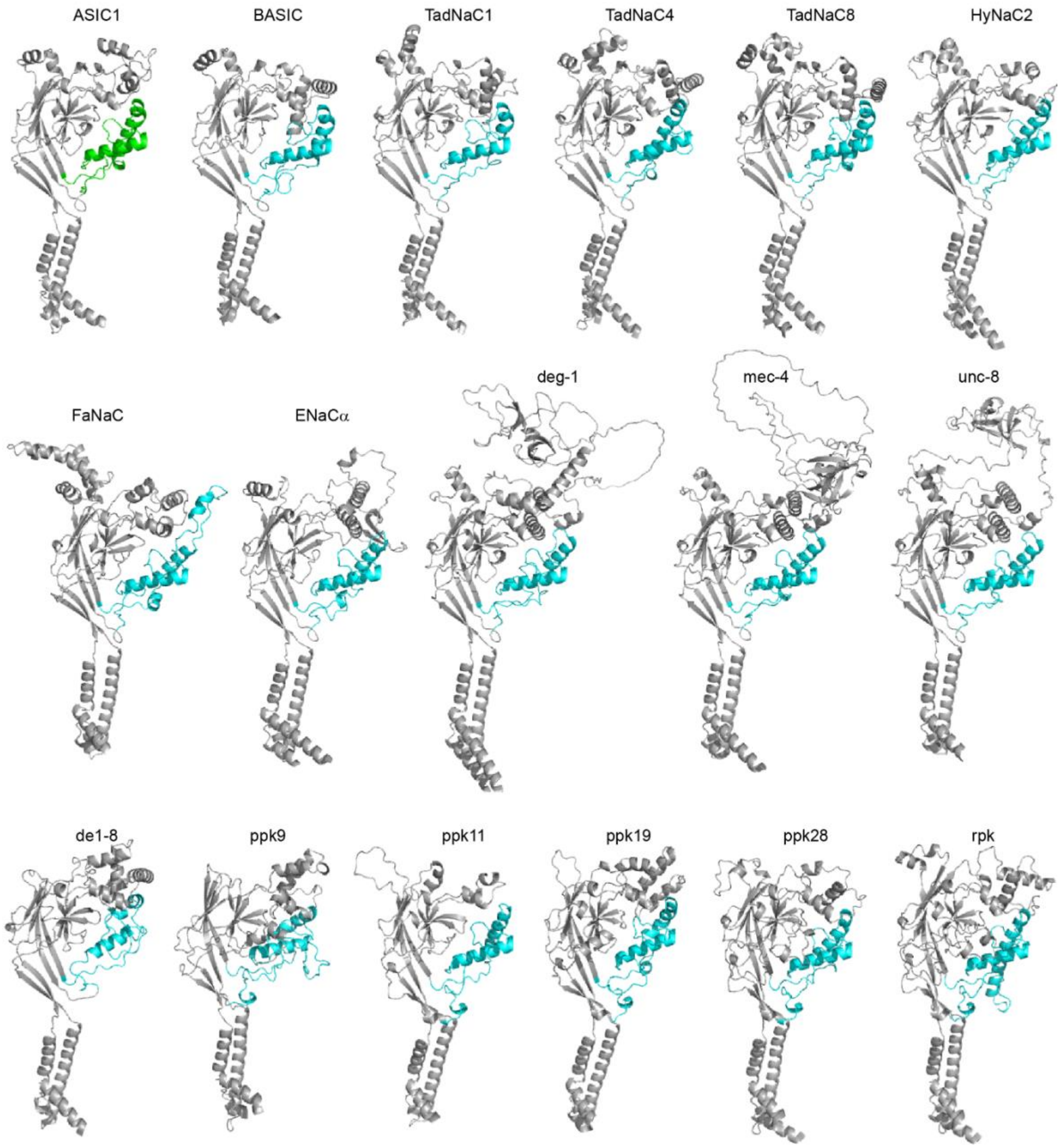

Figure S7: Structural conservation of the thumb domain across the degenerin/epithelial sodium channel (DEG/ENaC) family. AlphaFold 3 models of proteins listed in Figure S6, illustrating the structural conservation of the overall thumb domains. The thumb domain sequence is highlighted in green for cASIC1a and in cyan for other channels.

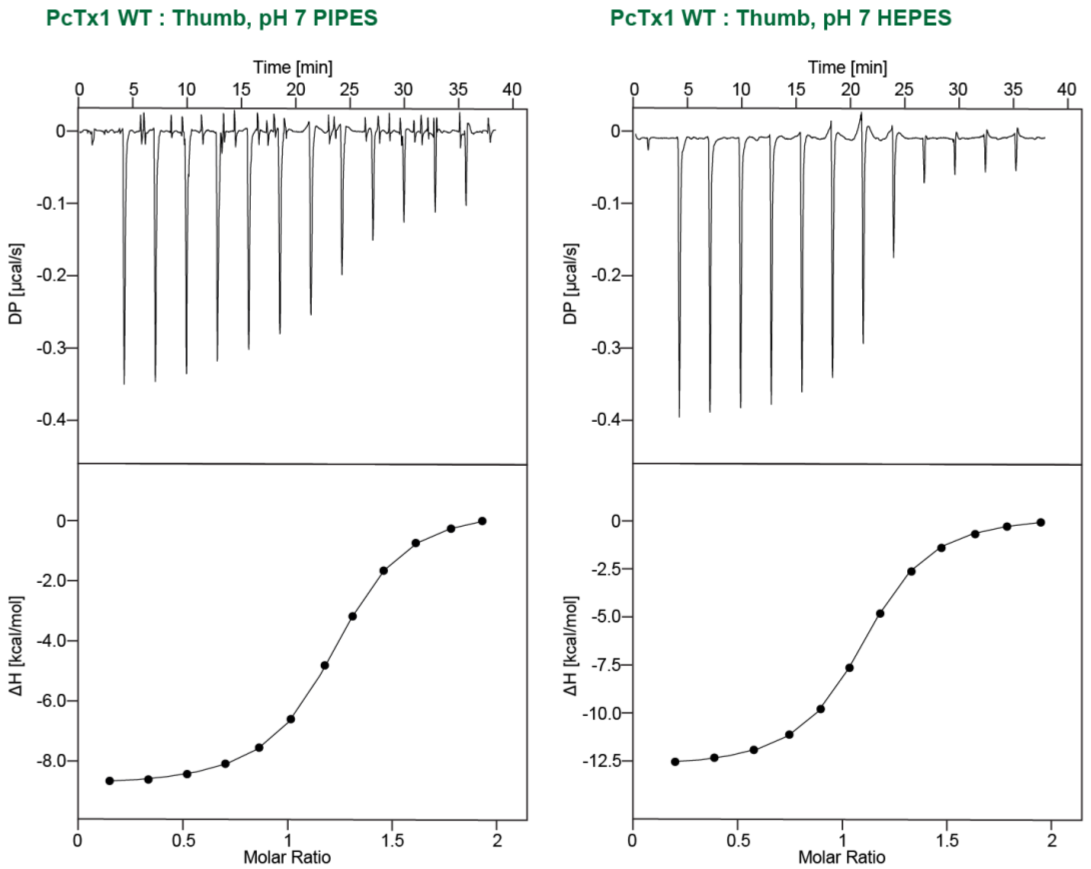

Figure S8: Example ITC traces for PcTx1 against the cASIC1a thumb at pH 7 in buffers with different ionisation enthalpies. See Table S4 for full thermodynamic parameters.

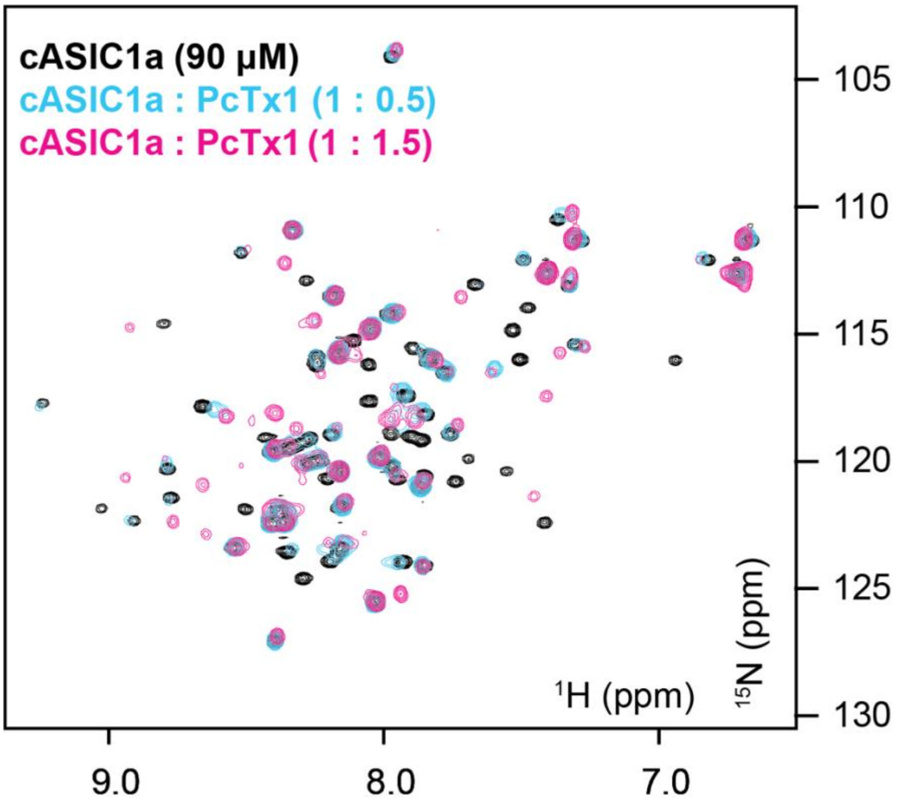

Figure S9: Titration of PcTx1 to ^{15}N-cASIC1a thumb by NMR. The spectra show evidence of slow exchange, consistent with nM binding observed by ITC (black, ^{15}N-cASIC1a thumb only [90 µM]; cyan, sub-saturating PcTx1 addition at a ratio of 1:0.5; magenta, saturating PcTx1 concentration at a ratio of 1:1.5). The bound state, at saturating PcTx1 concentration (magenta), shows extensive chemical shift changes.

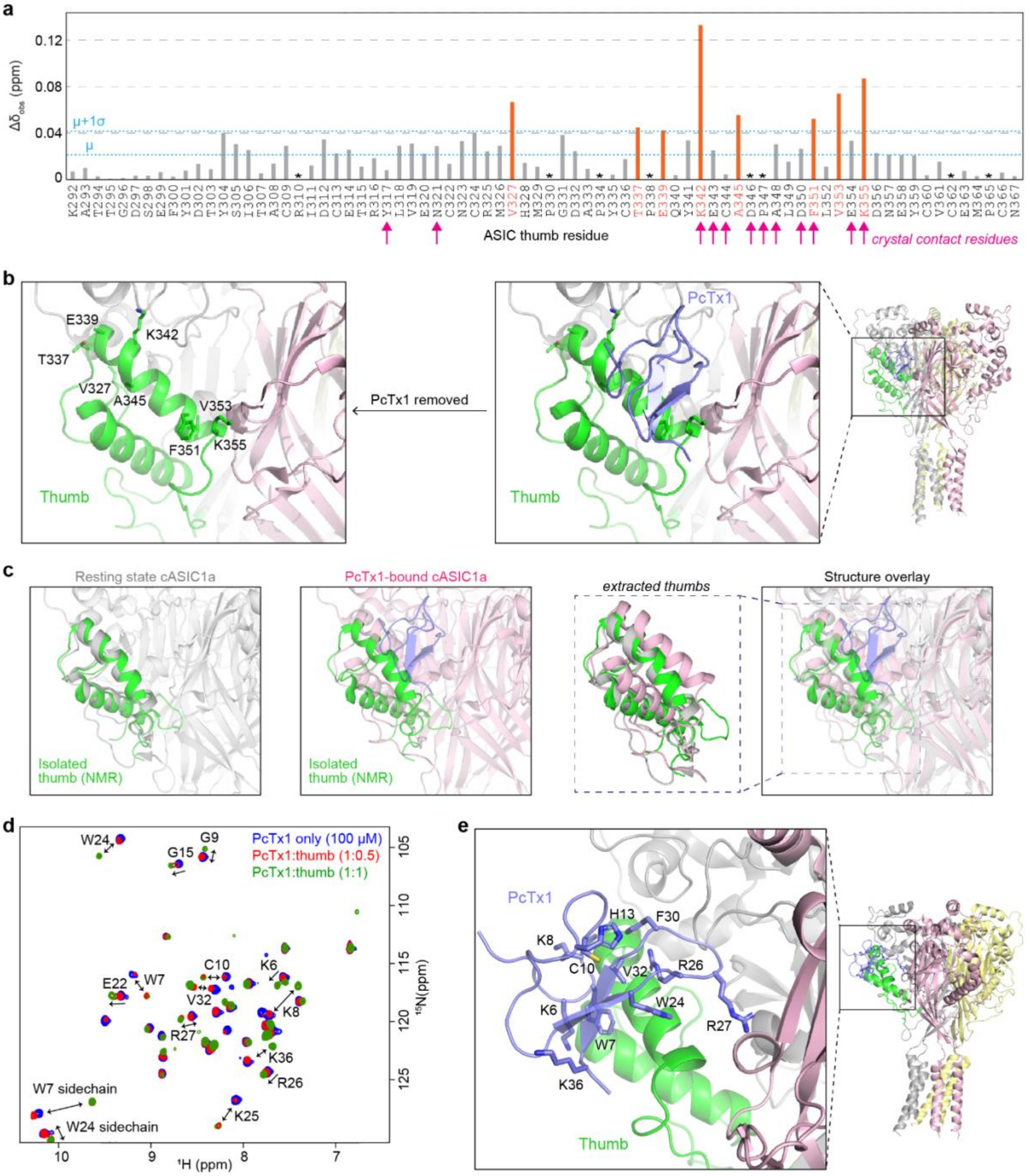

Figure S10: Mapping the binding interface between PcTx1 and cASIC1a by NMR. **a** Histogram of chemical shift perturbations in cASIC1a thumb residues during titration with PcTx1 at sub-saturating concentration (1:0.5 of cASIC1a to PcTx1). The ^{15}N-cASIC1a thumb residues that showed considerable chemical shift changes are V327, T337, K342, A345, F351, V353, and K355. Orange bars and residue labels represent significant chemical shift changes (greater than mean + one standard deviation). Asterisks (*) indicate unobservable signals in the ^{1}H-^{15}N HSQC spectrum. Pink arrows indicate cASIC1a residues that are making interactions with PcTx1 in the co-crystal structure.[13] **b** Structure of the PcTx1-cASIC1a complex (PDB: 4FZ0), with side chains

shown as sticks mapping the thumb residues that underwent significant chemical shift changes during the NMR titration with PcTx1. **c** Structural comparison of the ASIC thumb domain between resting and PcTx1-bound states. Left: Overlay of the NMR structure of the isolated cASIC1a thumb (green, PDB: 7LIE) with the resting-state full-length channel (grey, PDB: 5WKU), showing high global structural similarity. Middle: Overlay of the isolated thumb (green) with the PcTx1-bound ASIC1a thumb (pink, PDB: 4FZ0), highlighting differences in conformation of the thumb domain induced by PcTx1 binding. Right: Extracted thumb domains from resting-state (grey), PcTx1-bound (pink), and isolated thumb (green) structures, showing the global rearrangement of the thumb domain upon PcTx1 interaction. **d** ^{15}N-PcTx1 chemical shifts in the ^{1}H-^{15}N HSQC spectra were monitored upon addition of unlabelled cASIC1a thumb, showing binding in the slow exchange regime, consistent with the high nanomolar K_D measured by ITC. Significant chemical shift changes were observed in PcTx1 residues K6, W7, K8, G9, C10, H14, G15, W24, R26, R27, F30, V32, and K36. **e** Structure of the PcTx1-cASIC1a complex (PDB: 4FZ0), highlighting key PcTx1 residues that showed significant chemical shift changes.

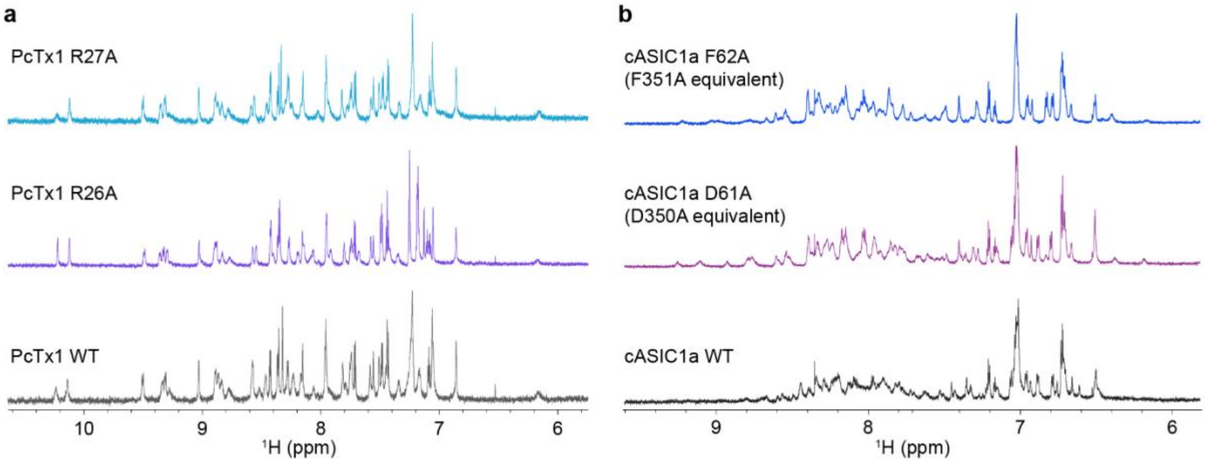

Figure S11: PcTx1 and c1a thumb mutants do not perturb the overall fold relative to their wild-type counterpart. a Comparison of the downfield region of the 1D ^{1}H NMR spectra of PcTx1 and mutants. **b** Comparison of the downfield region of the 1D ^{1}H NMR spectra of cASIC1a thumb and mutants.

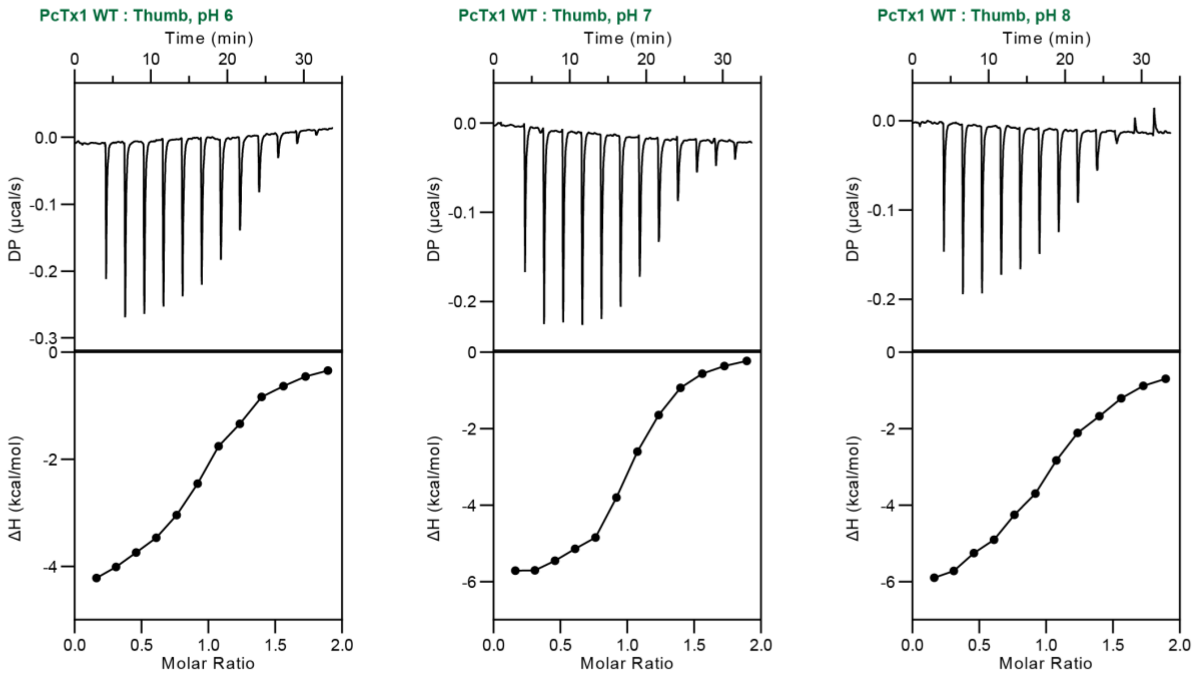

Figure S12: Example ITC traces for PcTx1 against the cASIC1a thumb at different pH conditions. See Table S6 for full thermodynamic parameters.

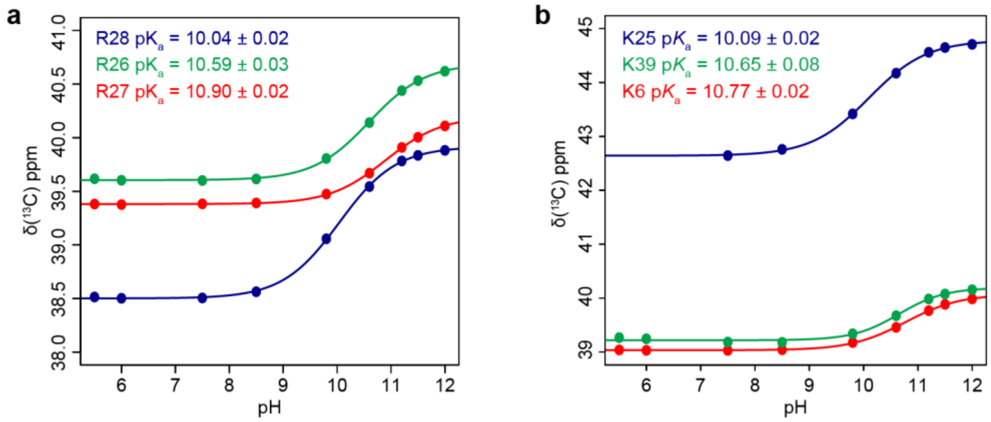

Figure S13: p*K*a determination of arginine and lysine side chains in PcTx1. a Arginine and **b** lysine ^{13}C chemical shifts of PcTx1 side chains at 900 MHz and 25 °C plot against experimental pH to calculate p*K*a values by fitting the Henderson-Hasselbalch equation.

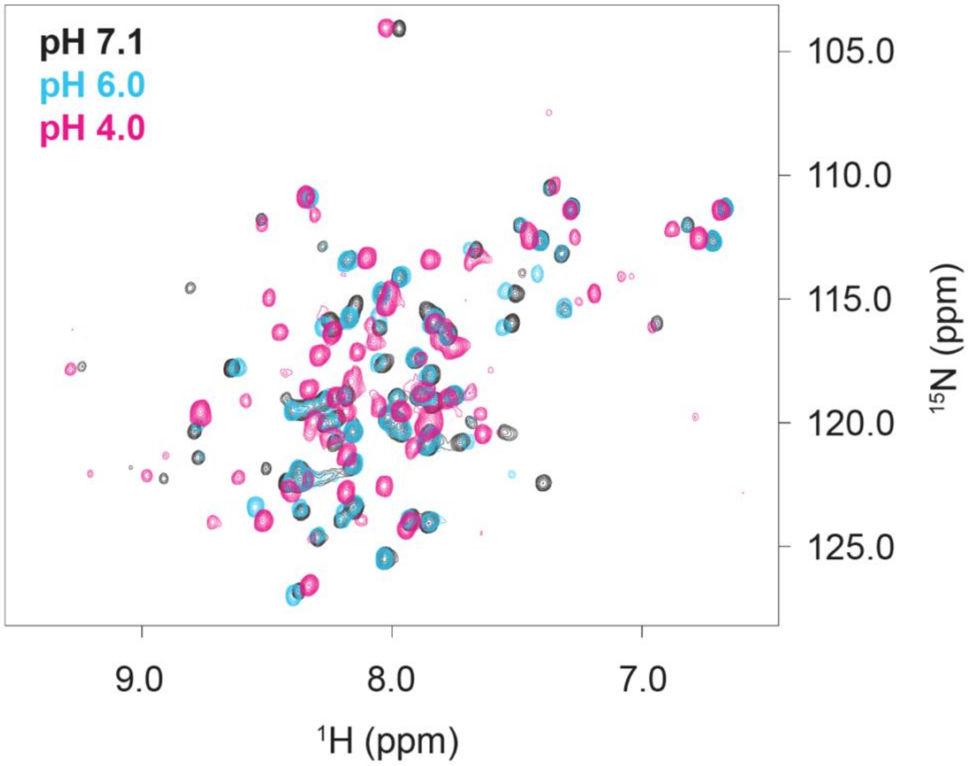

Figure S14: pH titration of ^{15}N-cASIC1a thumb by NMR. The spectra show large chemical shift changes for many residues between pH 6 and 4 indicative of significant structural changes.

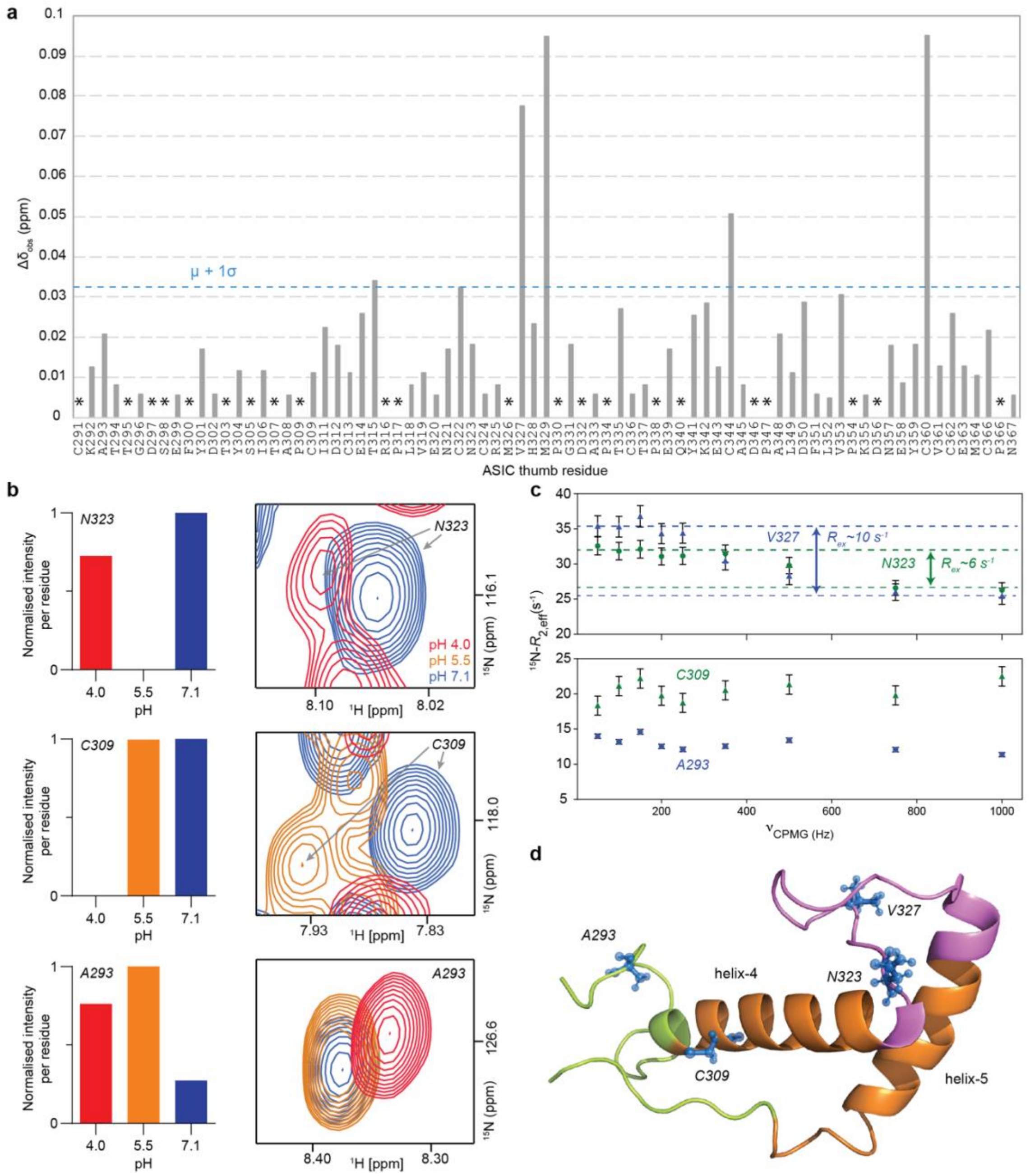

Figure S15: NMR chemical shift mapping of the cASIC1a thumb domain reveals pH-dependent chemical exchange. a Plot of chemical shift changes ($\Delta\delta$) due to a drop in pH from 7.1 to 6.0 vs cASIC1a thumb residue numbers. Asterisk indicates unobservable signals in the ^{1}H-^{15}N HSQC spectrum at one of the pHs. **b** Changes in peak intensity as a function of pH, examples of the three types of peak intensity changes observed. **c** ^{15}N CPMG relaxation dispersion profiles for selected residues at pH 6. Residues V327 and N323 show clear dispersion, indicative of μs–ms conformational exchange. Error bars based on spectral noise and replicate variation. Dashed lines indicate estimates of R_2^{eff} at low and high ν_{CPMG} limits (shaded), with the difference defining the dispersion amplitude (R_{ex}). **d** Mapping of three different regions on the thumb domain that correlate with the three pH-dependent peak intensity changes observed. Magenta shows regions in intermediate exchange at pH 5.5,

orange shows residues in intermediate exchange at pH 4, and green shows peaks that show exchange broadening at neutral pH.

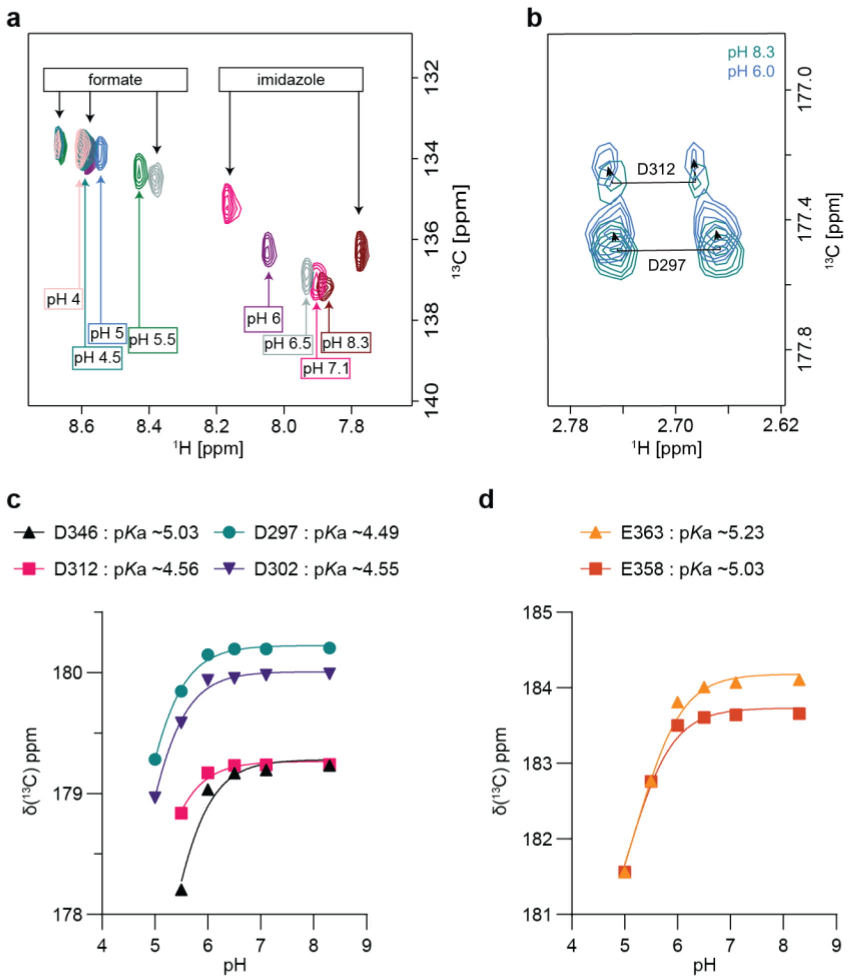

Figure S16: pH-sensitivity of protonatable side chains of the cASIC1a thumb domain. a ^{1}H-^{13}C HSQC spectra overlaid in a range of pH 4.0–8.3, where the exact pH was determined by monitoring the ^{1}H NMR spectrum of the sample using buffer components as internal pH indicators. **b** Superimposed 2D projections (^{1}H and carboxyl ^{13}C dimensions) from cASIC1a thumb aspartate peaks of 3D HCaCO experiments at two different pH values: pH 8.3 in teal, pH 6.0 in blue. **c,d** Carboxyl ^{13}C chemical shifts and experimental titration curves for **c** aspartate and **d** glutamate across a range of experimental pHs where only partial data was obtained for fitting of pK_a values, assuming a chemical shift change of 4 ppm upon protonation.

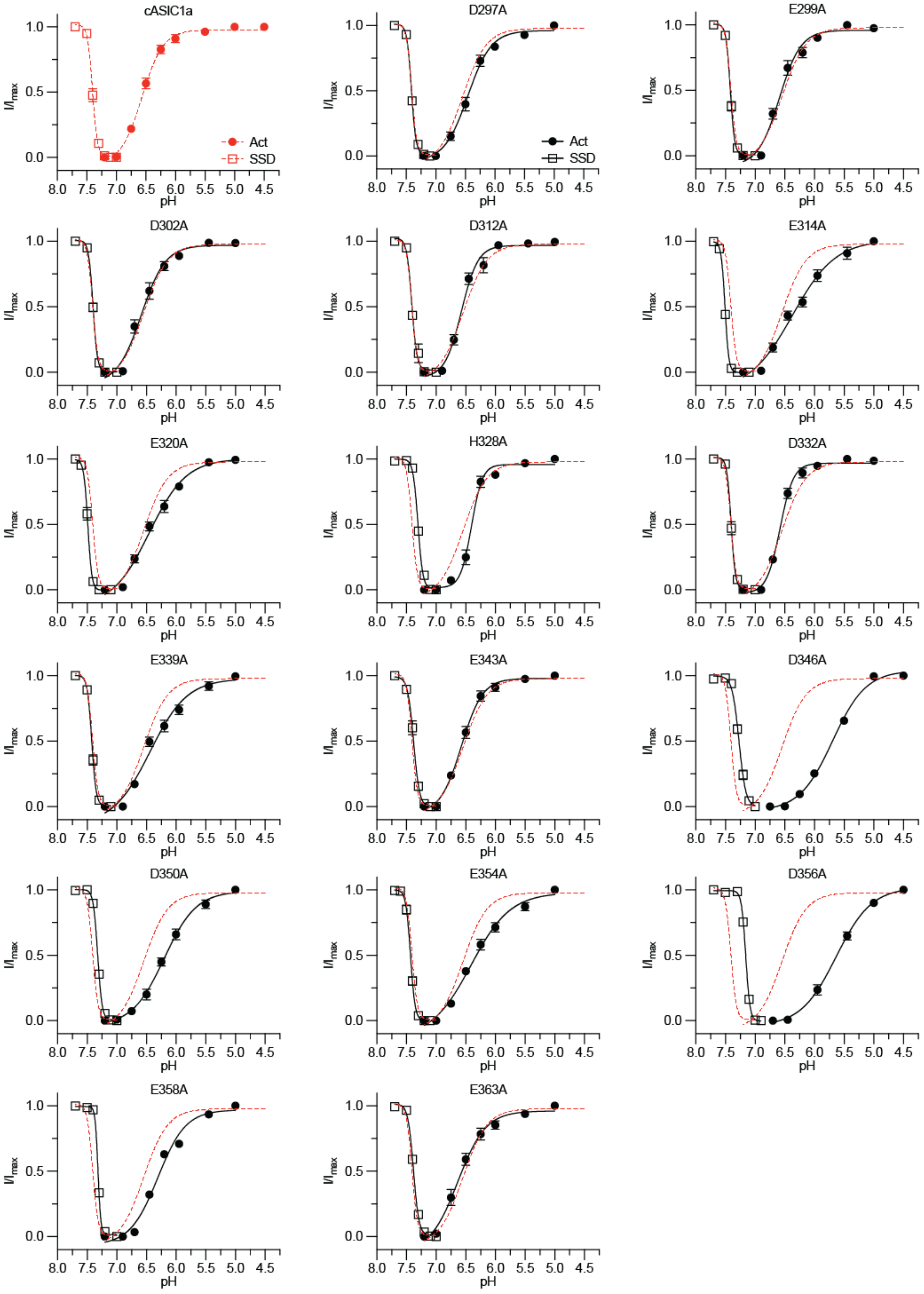

Figure S17: Electrophysiological determination of activation and steady-state desensitisation curves for cASIC1a wild-type and alanine mutants. Red traces in

each graph are the curve fit for cASIC1a wild-type as reference, and mutant traces are in black. Data are mean ± standard error of the mean, n = 6–9 independent oocytes, and fits of data are shown in Table S8.

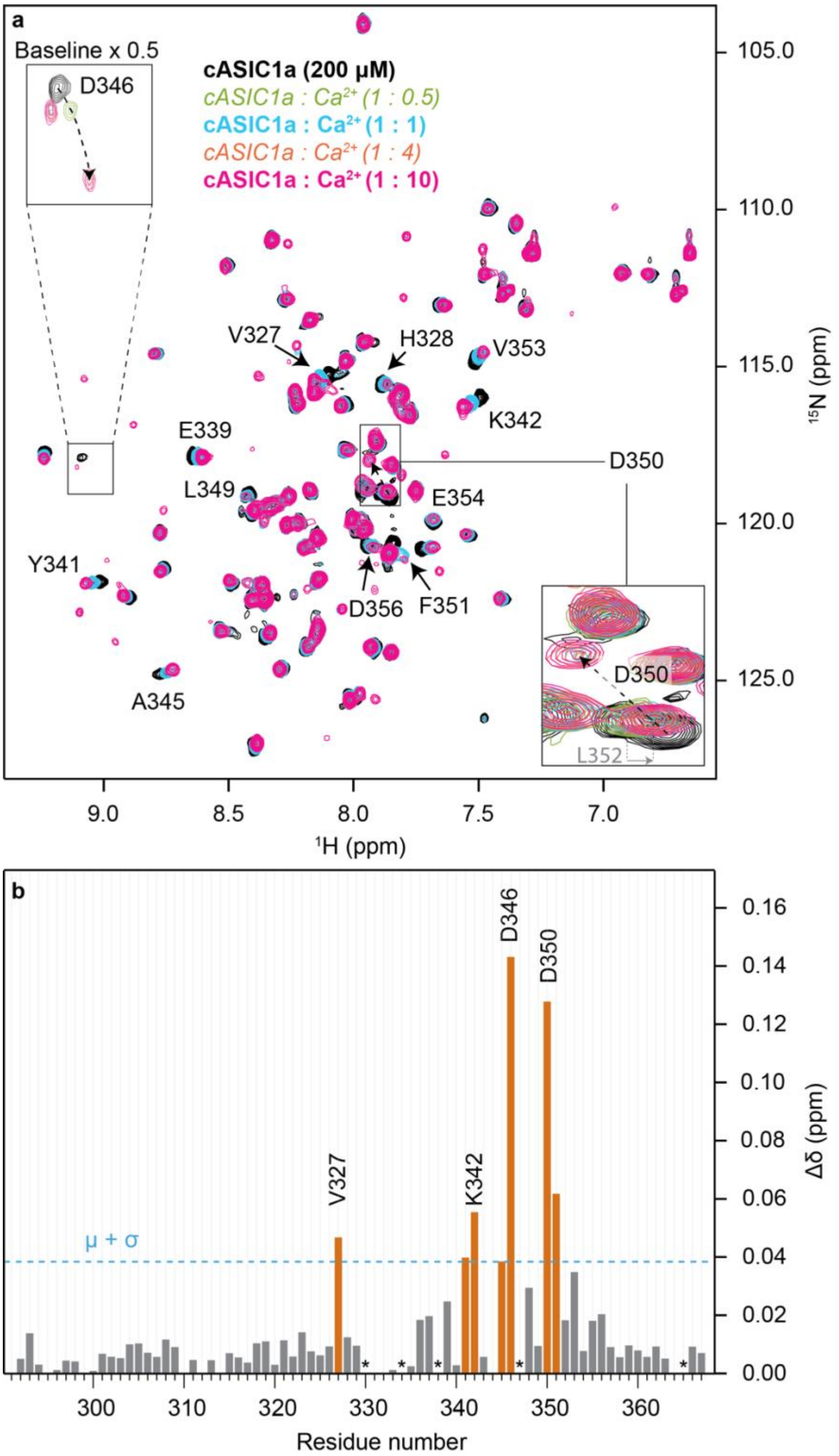

Figure S18: Chemical shift mapping of Ca^{2+} titration against ^{15}N-cASIC1a thumb. **a.** Overlay of ^{1}H-^{15}N HSQC spectra of cASIC1a thumb in presence of increasing Ca^{2+} concentration. **b** Plot of chemical shift changes ($\Delta\delta$) in presence and absence of 2 mM Ca^{2+}. Asterisk indicates unobservable signals in one of the ^{1}H-^{15}N HSQC spectra. All data in 50 mM Bis-Tris buffer at pH 7.

Supporting Tables

Table S1: Experimental parameters for NMR data acquired for structural characterisation of the cASIC1a thumb. The number of points in each dimension is given as sum of real and imaginary components, whereas number of NUS coordinates includes all four hypercomplex components.

Experiment	T1 (nucleus, points, time, carrier)	T2 (nucleus, points, time, carrier)	T3 (nucleus, points, time, carrier)	NUS / mixing time
2D ^{1}H-^{15}N H HSQC	**T1**: ^{15}N,	**T2**: ^{1}H,	–	-
	256 pts,	2048 pts,		
	23.4 ms,	69.6 ms,		
	115.5 ppm	4.77 ppm		
3D HNCO	**T1**: ^{13}C,	**T2**: ^{15}N,	**T3**: ^{1}H,	NUS (150)
	128 pts,	100 pts,	2048 pts,	
	17.7 ms,	20.3 ms,	81.9 ms,	
	173.5 ppm	115.5 ppm	4.77 ppm	
3D CBCA(CO)NH	**T1**: ^{13}C,	**T2**: ^{15}N,	**T3**: ^{1}H,	NUS (400)
	160 pts,	100 pts,	2048 pts,	
	4.4 ms,	17.1 ms,	81.9 ms,	
	43 ppm	115.5 ppm	4.77 ppm	
3D HNCACB	**T1**: ^{13}C,	**T2**: ^{15}N,	**T3**: ^{1}H,	NUS (600)
	200 pts,	100 pts,	2048 pts,	
	5.5 ms,	17.1 ms,	81.9 ms,	
	43 ppm	115.5 ppm	4.77 ppm	
3D HBHA(CO)NH	**T1**: ^{1}H,	**T2**: ^{15}N,	**T3**: ^{1}H,	NUS (500)
	128 pts,	90 pts,	2048 pts,	
	10.0 ms,	18.2 ms,	69.6 ms,	
	4.77 ppm	115.5 ppm	4.77 ppm	
3D C(CO)NH	**T1**: ^{13}C,	**T2**: ^{15}N,	**T3**: ^{1}H,	NUS (800)
	200 pts,	100 pts,	2048 pts,	
	4.9 ms,	17.1 ms,	69.6 ms,	
	43 ppm	115.5 ppm	4.77 ppm	
3D H(CO)NH	**T1**: ^{13}C,	**T2**: ^{15}N,	**T3**: ^{1}H,	NUS (900)
	160 pts,	100 pts,	2048 pts,	
	9.9 ms,	17.1 ms,	69.6 ms,	
	43 ppm	115.5 ppm	4.77 ppm	
3D ^{13}C edited NOESY (aliphatic region)	**T1**: ^{1}H,	**T2**: ^{13}C,	**T3**: ^{1}H,	Mixing = 120 ms
	140 pts,	80 pts,	2048 pts,	
	7.7 ms,	2.8 ms,	81.9 ms,	
	4.77 ppm	39 ppm	4.77 ppm	
	**T1**: ^{1}H,	**T2**: ^{13}C,	**T3**: ^{1}H,	

3D ^{13}C-edited NOESY (aromatic region)	80 pts,	60 pts,	2048 pts,	Mixing = 120 ms
	4.4 ms,	4.4 ms,	81.9 ms,	
	4.77 ppm	125 ppm	4.77 ppm	
3D ^{15}N edited NOESY	**T1**: ^{1}H,	**T2**: ^{13}C,	**T3**: ^{1}H,	Mixing = 120 ms
	120 pts,	74 pts,	2048 pts,	
	5.6 ms,	15 ms,	81.9 ms,	
	4.77 ppm	115.5 ppm	4.77 ppm	
2D ^{1}H-^{13}C HSQC	**T1**: ^{13}C,	**T2**: ^{1}H,	–	-
	256 pts,	2048 pts,		
	6.3 ms,	86.0 ms,		
	45 ppm	4.77 ppm		

Table S2: NMR and refinement statistics for 20 structures of the isolated cASIC1a thumb domain.

PDB ID: 7LIE	
BMRB ID: 30850	
**Experimental restraints**<br>Inter-proton distance restraints	
Intra-residue	329
Sequential	434
Medium-range (i–j < 5)	239
Long-range (i–j > 5)	141
Dihedral-angle restraints	121 (ϕ = 58, ψ = 63)
Disulfide-bond restraints	15
Total number of restraints per residue	16.4
**RMSD from mean coordinate structure (Å)**	
Backbone atoms (residues 305-354)	0.59 ± 0.15
All heavy atoms (residues 305-354)	0.94 ± *0.15*
**Stereochemical quality according to MolProbity[14]**	
Resides in most favoured Ramachandran region (%)	77.5
Ramachandran outliers (%)	1.65 ± 0.94
Unfavourable sidechain rotamers (%)	11.83 ± 2.79
Clashscore, all atoms	14.42 ± 2.21
Overall MolProbity score	3.10 ± 0.13

The clash score is the number of steric overlaps > 0.4 Å per 10^3 atoms. Statistics are mean ± standard deviation.

Table S3: Thermodynamic parameters for BigDyn and DynA 2–17 against cASIC1a thumb

Sample	K_D (μM)	ΔH (kcal/mol)	-TΔS (kcal/mol)	ΔG (kcal/mol)	N
BigDyn:Thumb	3.68 ± 0.48	-0.22 ± 0.01	-7.19 ± 0.40	-7.41 ± 0.51	0.95 ± 0.01
DynA 2–17:Thumb	2.22 ± 0.34	4.17 ± 0.25	-11.91 ± 1.66	-7.41 ± 1.91	1.12 ± 0.01

Data are mean ± standard error of the mean.

Table S4: Thermodynamic parameters for PcTx1 and cASIC1a thumb interactions from ITC measurements.

Sample	K_D (nM)	ΔH (kcal/mol)	-TΔS (kcal/mol)	ΔG (kcal/mol)	N
PcTx1:cASIC1a thumb	352.51 ± 48.23	-6.61 ± 0.64	-2.42 ± 0.15	-9.03 ± 0.79	1.07 ± 0.04
PcTx1:cASIC1a thumb, pH 7, HEPES	291.00 ± 8.49	-12.50 ± 0.42	3.59 ± 0.49	-8.93 ± 0.02	0.98 ±0.02
PcTx1:cASIC1a thumb, pH 7, PIPES	274.50 ± 4.95	-9.28 ±0.63	0.33 ± 0.64	-8.96 ±0.01	1.08 ± 0.05
PcTx1 R26A:Thumb WT	706.70 ± 23.28	-3.91 ± 0.56	-4.48 ± 0.77	-8.40 ± 0.33	1.03 ± 0.02
PcTx1 R27A:Thumb WT	553.00 ± 33.41	-8.19 ± 0.89	-0.35 ± 0.03	-8.54 ± 0.92	1.04 ± 0.03
PcTx1 WT:Thumb D350A	335.70 ± 31.22	-6.64 ± 0.72	-2.20 ± 0.24	-8.84 ± 0.96	1.01 ± 0.06
PcTx1 WT:Thumb F351A	no binding				

All data in 50 mM Citrate-Phosphate buffer at pH 7 unless otherwise stated. Data are mean ± standard error of the mean.

Table S5: Concentration-response parameters for PcTx1 WT and mutants agonist and inhibitory activity at cASIC1a WT and mutants

Sample	EC_{50} or IC_{50} (nM)	pEC_{50} or pIC_{50}	Fold change relative to PcTx1 WT:cASIC1a WT
Agonist activity at pH 7.45			
PcTx1 WT:cASIC1a WT	95.14	7.02 ± 0.10	–
PcTx1 R26A:cASIC1a WT*	>10,000	<5	>100
PcTx1 R27A:cASIC1a WT	5021.30	5.30 ± 0.24	52.77
PcTx1 WT:cASIC1a D350A	55.23	7.26 ± 0.10	0.58

PcTx1 WT:cASIC1a F351A*	>3000	<5.5	>30
Inhibition of pH 5-evoked current			
PcTx1 WT:cASIC1a WT	12.41	7.91 ± 0.09	–
PcTx1 R26A:cASIC1a WT*	>3000	<5.5	>240
PcTx1 R27A:cASIC1a WT	1480.00	5.83 ± 0.08	119.26
PcTx1 WT:cASIC1a D350A	14.61	7.91 ± 0.09	1.18
PcTx1 WT:cASIC1a F351A*	>3000	<5.5	>240

* Where full curve fitting was not possible due to the absence of higher-concentration data points, minimum IC_{50} and pIC_{50} values are reported to allow comparison across variants. Data are mean ± standard error of the mean where applicable.

Table S6: Thermodynamic parameters for PcTx1 binding to the cASIC1a thumb at different pH values.

pH	K_D (nM)	ΔH (kcal/mol)	-TΔS (kcal/mol)	ΔG (kcal/mol)	N
**PcTx1**					
5.5	1227.23 ± 127.20	-7.13 ± 0.74	-0.94 ± 0.12	-8.07 ± 0.86	1.07 ± 0.98
6.0	1000.71 ± 150.00	-6.33 ± 0.71	-1.84 ± 0.13	-8.17 ± 0.84	1.06 ± 0.93
6.5	375.00 ± 89.51	-8.18 ±0.67	-0.73 ± 0.07	-8.91 ± 0.73	1.20 ± 0.18
7.0	352.51 ± 48.23	-6.61 ± 0.64	-2.42 ± 0.15	-9.03 ± 0.79	1.07 ± 0.04
7.5	1433.15 ± 58.97	-8.01 ± 0.65	-1.10 ± 0.09	-9.10 ± 0.74	1.03 ± 0.07
8.0	1437.59 ± 161.10	-7.82 ± 0.78	-0.47 ± 0.05	-8.29 ± 0.83	1.06 ± 0.04

Data are mean ± standard error of the mean.

Table S7: Normalised NMR peak intensities at different pH values.

Residue	pH 7.1	pH 6.5	pH 6.0	pH 5.5	pH 5.0	pH 4.5	pH 4.0	pH 3.5	pH 3.0
**C291**	1.000	*	*	*	*	*	*	*	*
**K292**	0.089	0.297	0.452	1.000	0.840	0.766	0.721	0.568	0.219
**A293**	0.274	0.403	0.505	1.000	0.790	0.734	0.761	0.578	0.240
**T294**	0.255	0.426	0.520	1.000	0.819	0.769	0.786	0.582	0.269
**T295**	0.236	0.400	0.524	1.000	0.981	0.951	0.917	0.719	0.328
**G296**	0.128	0.328	0.467	1.000	0.777	0.716	0.671	0.546	0.247
**D297**	0.350	0.501	0.585	1.000	0.746	0.756	0.660	0.559	0.256

**S298**	0.373	0.511	0.578	1.000	0.851	0.783	0.752	0.647	0.268
**E299**	0.895	0.928	0.804	1.000	0.579	0.627	0.709	0.700	0.263
**F300**	0.660	0.678	0.637	1.000	0.624	0.401	0.396	0.328	0.162
**Y301**	0.334	0.812	0.727	0.567	0.450	0.972	1.000	0.864	0.365
**D302**	0.730	0.798	0.729	1.000	0.541	0.659	0.667	0.578	0.253
**T303**	0.509	0.628	0.637	1.000	0.596	0.752	0.803	0.711	0.315
**Y304**	1.000	0.999	0.575	0.827	0.478	0.641	0.747	0.680	0.293
**S305**	0.825	1.000	0.830	0.998	0.378	0.323	0.477	0.454	0.179
**I306**	0.743	0.797	1.000	0.795	0.660	*	*	*	*
**T307**	0.909	0.762	0.666	1.000	0.660	0.753	0.971	0.888	0.357
**A308**	0.855	0.753	0.581	0.643	0.235	0.659	1.000	0.658	0.228
**C309**	1.000	0.988	0.760	0.996	0.435	*	*	*	*
**R310**	0.786	0.774	0.412	1.000	0.384	*	*	*	*
**R310 (He,Ne)**	0.562	0.752	0.779	1.000	0.654	0.778	0.691	0.955	0.371
**I311**	0.522	0.606	0.473	0.740	0.612	1.000	0.290	0.263	0.120
**D312**	0.617	0.771	1.000	0.822	0.460	0.666	0.623	0.627	0.219
**C313**	0.470	0.364	0.360	1.000	0.363	0.550	0.598	0.457	0.239
**E314**	0.246	0.246	0.132	0.336	0.745	0.955	1.000	0.767	0.335
**T315**	0.791	0.755	0.795	1.000	0.641	0.664	0.705	0.514	0.238
**R316**	1.000	0.835	0.573	0.809	0.109	*	*	*	*
**R316 (He,Ne)**	0.357	0.526	0.578	0.998	0.815	1.000	0.877	0.696	0.316
**Y317**	1.000	0.817	0.845	0.660	0.746	*	*	*	*
**L318**	0.736	0.679	0.248	0.778	*	0.816	1.000	0.847	0.335
**V319**	1.000	0.913	0.416	*	*	0.477	0.583	*	*
**E320**	1.169	0.812	0.651	0.825	0.577	*	*	*	*
**N321**	0.935	0.748	0.566	0.940	0.686	0.993	1.000	0.848	0.423
**N321 (Hd2a, Nd2)**	0.725	0.823	0.258	*	*	0.790	0.735	1.000	0.389
**N321 (Hd2b, Nd2)**	0.820	0.483	*	*	*	0.974	1.000	0.863	0.431
**C322**	0.491	0.327	0.199	*	*	0.882	1.000	0.766	0.390
**N323**	1.000	0.711	0.591	*	*	*	0.727	0.667	0.376
**N323 (Hd2b, Nd2)**	0.703	0.674	0.562	1.000	0.892	0.880	0.796	0.712	0.301
**N323 (Hd2a, Nd2)**	0.607	0.546	0.511	1.000	0.784	0.823	0.779	0.622	0.146
**C324**	1.000	0.801	0.518	0.722	0.557	0.886	0.963	0.810	0.408
**R325**	0.845	0.682	0.484	0.789	0.561	0.724	1.000	0.765	0.288
**R325 (He,Ne)**	1.000	0.817	0.449	0.969	0.983	0.970	0.832	0.727	0.264
**M326**	0.947	0.612	0.300	*	*	0.624	1.000	0.883	0.264
**V327**	1.000	0.975	0.556	0.578	*	*	*	*	*
**H328**	0.484	0.295	0.277	0.731	0.917	0.965	1.000	0.711	0.293

**M329**	1.000	0.497	0.241	*	*	*	*	*	*
**G331**	0.739	0.505	0.167	*	*	0.696	1.000	0.582	0.265
**D332**	1.000	0.707	0.478	0.737	0.623	0.729	0.746	0.651	0.336
**A333**	0.787	0.583	0.482	0.947	0.751	0.954	1.000	0.696	0.299
**Y335**	0.750	0.671	0.598	0.877	0.756	0.624	1.000	0.885	0.333
**C336**	0.716	0.579	0.332	*	*	0.755	1.000	0.867	*
**T337**	0.655	0.431	*	*	0.637	0.976	1.000	0.786	0.391
**E339**	0.690	0.590	0.498	0.944	0.851	1.000	0.983	0.788	0.277
**Q340**	0.676	0.509	0.549	1.000	0.877	0.925	0.810	0.642	0.283
**Q340 (He2b,Ne2)**	0.672	0.531	0.517	1.000	0.711	0.854	0.807	0.785	0.293
**Q340 (He2a,Ne2)**	0.720	0.558	0.434	0.767	0.673	0.696	1.000	0.642	0.710
**Y341**	0.704	0.684	*	0.147	*	0.845	0.954	1.000	0.353
**K342**	0.502	0.413	0.304	0.605	0.570	0.704	1.000	0.883	0.421
**E343**	0.750	0.612	0.342	0.068	0.445	0.472	1.000	0.983	0.400
**C344**	1.000	0.706	0.617	0.967	0.663	0.641	0.677	0.652	0.221
**A345**	1.000	0.824	*	*	*	*	*	*	0.780
**D346**	1.000	*	*	*	*	*	*	*	*
**A348**	0.688	0.491	0.324	0.531	0.384	0.747	1.000	0.848	0.341
**L349**	0.223	0.172	0.371	1.000	0.868	0.656	0.638	0.540	0.227
**D350**	0.948	0.879	0.761	0.715	0.649	0.816	1.000	0.806	0.341
**F351**	0.849	0.629	0.524	0.482	0.692	1.000	0.949	0.622	0.235
**L352**	0.687	0.576	0.620	1.000	0.642	*	*	*	*
**V353**	1.000	0.778	0.601	0.766	0.497	*	*	*	*
**E354**	0.736	0.531	0.335	1.000	0.686	0.727	0.687	0.690	0.251
**K355**	0.979	0.934	0.589	1.000	0.570	*	0.316	0.520	0.289
**D356**	0.421	0.474	0.527	1.000	0.335	*	*	*	*
**N357**	0.873	0.739	0.585	0.913	0.474	0.821	1.000	0.744	0.289
**N357 (Hd2b,Nd2)**	0.866	0.751	0.515	1.000	0.722	0.842	0.679	0.575	0.004
**N357 (Hd2a,Nd2)**	0.765	0.800	0.533	1.000	*	0.157	*	*	*
**E358**	1.000	0.833	0.595	0.322	*	*	*	*	*
**Y359**	0.612	0.516	0.325	0.370	0.310	0.936	0.992	1.000	0.326
**C360**	0.824	1.000	*	*	0.087	*	0.261	0.260	0.004
**V361**	1.000	0.914	0.785	0.933	0.947	0.465	0.443	0.452	0.306
**C362**	1.000	0.901	0.783	0.915	0.528	*	*	*	*
**E363**	1.000	0.743	0.497	0.542	0.369	*	*	*	*
**M364**	0.635	0.559	0.467	1.000	0.732	0.824	0.720	0.603	0.225
**C366**	0.633	0.684	0.590	1.000	0.927	0.687	0.577	0.475	0.013
**N367**	0.638	0.562	0.481	1.000	0.899	0.845	0.733	0.621	0.239

**N367 (Hd2b,Nd2)**	0.583	0.542	0.526	1.000	0.905	0.795	0.763	0.661	0.300
**N367 (Hd2a,Nd2)**	0.641	0.584	0.539	1.000	0.890	0.836	0.794	0.605	0.280

*no observable peaks

Table S8: Summary of electrophysiology experiments examining pH-sensing residues on the thumb domain.

	**Activation**			**Steady-state desensitisation**		
	**pH_{50}**	**slope**	**P value**	**pH_{50}**	**slope**	**P value**
**cASIC1a**	6.54 ± 0.03	2.15 ± 0.23	n/a	7.41 ± 0.01	9.59 ± 0.98	n/a
**D297A**	6.46 ± 0.03	2.51 ± 0.37	0.0807	7.41 ± 0.01	11.31 ± 0.96	0.7076
**E299A**	6.59 ± 0.03	2.40 ± 0.31	0.9994	7.42 ± 0.01	12.06 ± 1.02	0.1684
**D302A**	6.59 ± 0.03	2.14 ± 0.30	>0.9999	7.40 ± 0.01	11.72 ± 1.00	>0.9999
**D312A**	6.57 ± 0.02	3.12 ± 0.39	>0.9999	7.41 ± 0.01	10.10 ± 1.56	0.9982
**E314A**	6.37 ± 0.06	1.20 ± 0.20	0.1588	7.51 ± 0.01	13.56 ± 1.16	**<0.0001**
**E320A**	6.45 ± 0.04	1.39 ± 0.17	0.3462	7.49 ± 0.01	12.70 ± 1.52	**<0.0001**
**H328A**	6.40 ± 0.02	4.68 ± 0.57	**0.0006**	7.31 ± 0.01	10.51 ± 0.77	**<0.0001**
**D332A**	6.58 ± 0.01	3.84 ± 0.34	>0.9999	7.40 ± 0.01	12.13 ± 1.61	>0.9999
**E339A**	6.43 ± 0.05	1.39 ± 0.20	0.3863	7.42 ± 0.01	11.60 ± 1.26	0.1033
**E343A**	6.57 ± 0.02	2.43 ± 0.26	>0.9999	7.38 ± 0.01	8.66 ± 0.86	0.0861
**D346A**	5.69 ± 0.02	1.55 ± 0.11	**<0.0001**	7.27 ± 0.01	7.57 ± 0.71	**<0.0001**
**D350A**	6.20 ± 0.03	1.61 ± 0.19	**<0.0001**	7.32 ± 0.01	11.70 ± 0.74	**<0.0001**
**E354A**	6.41 ± 0.04	1.31 ± 0.18	0.1330	7.43 ± 0.01	11.07 ± 0.79	**0.0029**
**D356A**	5.63 ± 0.03	1.45 ± 0.16	**<0.0001**	7.16 ± 0.01	12.05 ± 0.58	**<0.0001**
**E358A**	6.31 ± 0.02	1.85 ± 0.17	**<0.0001**	7.32 ± 0.01	18.33 ± 2.02	**<0.0001**
**E363A**	6.63 ± 0.05	1.84 ± 0.29	0.9032	7.38 ± 0.01	9.30 ± 0.58	**0.0227**

Data are mean ± standard error of the mean. P values are calculated compared to pH_{50} values from WT cASIC1a via Welch's one-way ANOVA with Dunnett's multiple comparisons test, and bold values indicates a significant difference ($P < 0.05$).

Supporting References

[1] aJ. K. Klint, S. Senff, N. J. Saez, R. Seshadri, H. Y. Lau, N. S. Bende, E. A. Undheim, L. D. Rash, M. Mobli, G. F. King, *PLoS One* **2013**, *8*, e63865; bN. J. Saez, B. Cristofori-Armstrong, R. Anangi, G. F. King, *Methods Mol Biol* **2017**, *1586*, 155-180.

[2] M. Mobli, J. C. Hoch, *Concepts Magn Reson Part A Bridg Educ Res* **2008**, *32A*, 436-448.

[3] M. Mobli, *J Magn Reson* **2015**, *256*, 60-69.

[4] J. C. S. Hoch, A. , *NMR Data Processing*, John Wiley & Sons Inc (US), **1996**.
[5] W. F. Vranken, W. Boucher, T. J. Stevens, R. H. Fogh, A. Pajon, M. Llinas, E. L. Ulrich, J. L. Markley, J. Ionides, E. D. Laue, *Proteins* **2005**, *59*, 687-696.
[6] Y. Shen, A. Bax, *J Biomol NMR* **2013**, *56*, 227-241.
[7] P. Guntert, *Methods Mol Biol* **2004**, *278*, 353-378.
[8] N. J. Saez, M. Mobli, M. Bieri, I. R. Chassagnon, A. K. Malde, R. Gamsjaeger, A. E. Mark, P. R. Gooley, L. D. Rash, G. F. King, *Mol Pharmacol* **2011**, *80*, 796-808.
[9] T. Yuwen, L. E. Kay, *J Biomol NMR* **2019**, *73*, 641-650.
[10] aB. Cristofori-Armstrong, E. Budusan, L. D. Rash, *Proc Natl Acad Sci U S A* **2021**, *118*; bB. Cristofori-Armstrong, M. S. Soh, S. Talwar, D. L. Brown, J. D. Griffin, Z. Dekan, J. L. Stow, G. F. King, J. W. Lynch, L. D. Rash, *Sci Rep* **2015**, *5*, 14763.
[11] L. Leisle, M. Margreiter, A. Ortega-Ramirez, E. Cleuvers, M. Bachmann, G. Rossetti, S. Grunder, *J Med Chem* **2021**, *64*, 13299-13311.
[12] C. B. Borg, N. Braun, S. A. Heusser, Y. Bay, D. Weis, I. Galleano, C. Lund, W. Tian, L. M. Haugaard-Kedstrom, E. P. Bennett, T. Lynagh, K. Stromgaard, J. Andersen, S. A. Pless, *Proc Natl Acad Sci U S A* **2020**, *117*, 7447-7454.
[13] R. J. Dawson, J. Benz, P. Stohler, T. Tetaz, C. Joseph, S. Huber, G. Schmid, D. Hugin, P. Pflimlin, G. Trube, M. G. Rudolph, M. Hennig, A. Ruf, *Nat Commun* **2012**, *3*, 936.
[14] I. W. Davis, A. Leaver-Fay, V. B. Chen, J. N. Block, G. J. Kapral, X. Wang, L. W. Murray, W. B. Arendall, 3rd, J. Snoeyink, J. S. Richardson, D. C. Richardson, *Nucleic Acids Res* **2007**, *35*, W375-383.
